# Human study on cancer diagnostic probe (CDP) for real‐time excising of breast positive cavity side margins based on tracing hypoxia glycolysis; checking diagnostic accuracy in non‐neoadjuvant cases

**DOI:** 10.1002/cam4.4503

**Published:** 2022-02-28

**Authors:** Zohreh Sadat Miripour, Fereshteh Abbasvandi, Parisa Aghaee, Fatemeh Shojaeian, Mahsa Faramarzpour, Pooneh Mohaghegh, Parisa Hoseinpour, Naser Namdar, Morteza Hassanpour Amiri, Hadi Ghafari, Mohammad Parniani, Ahmad Kaviani, Sedigheh Alamdar, Sahar NajafiKhoshnoo, Hassan Sanati, Mahna Mapar, Nastaran Sadeghian, Mohammad Esmaeil Akbari, Masud Yunesian, Mohammad Abdolahad

**Affiliations:** ^1^ Nano Bio Electronic Devices Lab School of Electrical and Computer Engineering College of Engineering University of Tehran Tehran Iran; ^2^ Nano Electronic Center of Excellence Thin Film and Nanoelectronics Lab School of Electrical and Computer Engineering College of Engineering University of Tehran Tehran Iran; ^3^ ATMP Department Breast Cancer Research Center Motamed Cancer Institute ACECR Tehran Iran; ^4^ Cancer Research Center Shahid Beheshti University of Medical Sciences Tehran Iran; ^5^ SEPAS Pathology Laboratory Tehran Iran; ^6^ Pathology Department Breast Cancer Research Center Motamed Cancer Institute ACECR Tehran Iran; ^7^ Institute of Cancer Imam Khomeini Hospital Tehran University of Medical Sciences Tehran Iran; ^8^ Department of Pathobiology Iran University of Medical Sciences Shahid Hemmat Highway Tehran Iran; ^9^ Department of Environmental Health School of Public Health Tehran University of Medical Sciences Tehran Iran; ^10^ Department of Research Methodology and Data Analysis Institute for Environmental Research Tehran University of Medical Sciences Tehran Iran; ^11^ UT&TUMS Cancer Electronics Research Center Tehran University of Medical Sciences Tehran Iran

**Keywords:** cancer surgery, clinical study, hypoxia assisted glycolysis, pathology

## Abstract

**Background:**

Cancer diagnostic probe (CDP) had been developed to detect involved breast cavity side margins in real‐time (Miripour et al. Bioeng Transl Med. e10236.). Here, we presented the results of the in vivo human model CDP studies on non‐neoadjuvant cases.

**Methods:**

This study is a prospective, blind comparison to a gold standard, and the medical group recruited patients. CDP and frozen data were achieved before the permanent pathology experiment. The main outcome of the study is surgical margin status. From November 2018 to April 2020, 202 patients were registered, and 188 were assigned for the study. Breast‐conserving surgery at any age or gender, re‐surgery due to re‐currency, or involved margins are acceptable. Patients must be non‐neoadjuvant. The reliability of CDP scoring had been evaluated by the pathology of the scored IMs. Then, three models of the study were designed to compare CDP with the frozen sections. Receiver operating characteristic (ROC) curves and AUC were measured based on the permanent postoperative pathology gold standard.

**Results:**

A matched clinical diagnostic categorization between the pathological results of the tested IMs and response peaks of CDP on 113 cases, was reported (sensitivity = 97%, specificity = 89.3%, accuracy = 92%, positive predictive value (PPV) = 84.2%, and negative predictive value (NPV) = 98%). Study A showed the independent ability of CDP for IM scoring (sensitivity = 80%, specificity = 90%, accuracy = 90%, PPV = 22.2%, and NPV = 99.2%). Study B showed the complementary role of CDP to cover the missed lesions of frozen sections (sensitivity = 93.8%, specificity = 91%, accuracy = 91%, PPV = 55.6%, and NPV = 99.2%). Study C showed the ability of CDP in helping the pathologist to reduce his/her frozen miss judgment (specificity = 92%, accuracy = 93%, PPV = 42.1%, and NPV = 100%). Results were reported based on the post‐surgical permanent pathology gold standard.

**Conclusion:**

CDP scoring ability in intra‐operative margin detection was verified on non‐neoadjuvant breast cancer patients. Non‐invasive real‐time diagnosis of IMs with pathological values may make CDP a distinct tool with handheld equipment to increase the prognosis of breast cancer patients.

## Graphical abstract

A real‐time system, named cancer diagnostic probe (CDP), had been developed to diagnose the presence of pre‐neoplastic/neoplastic cells in either internal or external margins of the patients during breast cancer surgery by lively determining the hypoxia assisted glycolysis pathways.

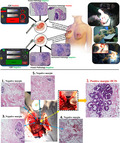

## 1 INTRODUCTION

Breast cancer (BC) is one of the most important tumor diseases, which makes it the world’s most prevalent women cancer in 2020 with significant mortality.^1^, ^2^ It could appear and progress in various phenotypes from luminal A to Basal and from lobular to ductal initiation.^3^ Early diagnosis of BC could be so helpful in the overall survival (OS) of the patients.^4^ Different guidelines of therapies were recommended for BC due to its stage, and phenotypes ranged from surgery to chemotherapy and radiotherapy.^5^, ^6^, ^7^, ^8^, ^9^ If the BC cases were a candidate for surgery as the first step of therapy (non‐neoadjuvant cases), breast‐conserving surgery (BCS) or mastectomy could be performed due to the stage of the disease. For example, a single invasive ductal carcinoma (IDC) tumor with a size smaller than 2 cm could be a candidate for BCS,^10^ while multifocal scattered ductal carcinoma in situ (DCIS) would be referred to mastectomy.^11^, ^12^

The main goal in conservative breast cancer surgery is to remove cancer tumors with safe margins intraoperatively. So, no involved lesions must be remained in the surgical field to prevent the second surgery. Remained positive margins not only may increase the local recurrence rate of breast tumor,^13^ but also the cancer cells in cavity side margins can be hyperactivated due to cytokine accumulation in tumor bed as an inflammatory ambient. Also, angiogenesis required for wound healing can prepare VEGF for cancer cells to be more progressive.^14^, ^15^ Hence, precise real‐time detection during the surgery is so crucial. Although many attempts and methods were assessed to achieve free cavity side margin (Table S1), reports indicated that up to 40% of the involved margins^16^, ^17^, ^18^, ^19^ still could not be diagnosed intra‐operatively by conventional intra‐operative methods such as frozen section^20^ and x‐ray evaluation^21^ of the margins in the dissected tumor. Other newly reported systems such as MassPen (protein spectroscopy of cavity side margin),^22^ Margin Probe (radio frequency spectroscopy),^23^ etc.^24^ were still in progress, and many clinical trials would be required to be ensured from their efficacy.

Cancer diagnostic probe (CDP) is a real‐time margin detection system based on finding hypoxia glycolysis of neoplastic breast cells in cavity side margins with pathological calibration by a handheld electrical tool^25^ (US Patent Pub. No. 10,786,188 B1). In comparison with some new research‐based margin detection technologies such as MassPen,^22^ Margin Probe,^23^ and confocal laser endomicroscopy (CLE)^26^ the distinct ability of CDP is real‐time checking of the cavity sidewall with a well‐known mechanism (hypoxia glycolysis) as well as pathological calibration for scoring. This can be used as a great complementary system near or maybe as an alternative procedure instead of a frozen section. Tumor side evaluation (even with slide preparation from the whole surface of the margin) might not provide reassurance about clearance of cavity side margins.^27^ Hence, CDP may shed new light in the future as an intra‐operative cavity side margin evaluating system. Here, we applied CDP in multi‐clinical studies to compare its efficacy with the frozen section.

In this paper, three clinical studies named A, B, and C evaluated the clinical efficacy of CDP during breast cancer surgery in non‐neoadjuvant cases.

Study A is observational in which the validation matching between CDP scores of IMs (internal margins on the cavity side) compared to the pathological evaluation of EMs (external margins on the tumor side) was studied. This study had no role in diagnosing and treating the patients. In this clinical study, CDP was applied for data recording from 150 margins of 25 human breast cancer cases without inducing any perturbation or intervention in the trend of conventional surgery.

Study B was an interventional study with registration ID: IRCT20190904044697N1. After margin shaving based on the frozen declaration, CDP was applied for data recording and sample re‐excision (from the exact location that CDP had positively scored with the volume of 3 × 3 × 4 mm^3^) from IMs of human cases of breast cancer. Hence, in study B, CDP has a complementary diagnostic role to re‐excise the probable remaining positive IMs missed in the frozen section of reciprocal EMs. Here, the gold standard was the permanent section of CDP samples (IMs).

Study C (registration ID: IRCT20190904044697N3) is an interventional trial applied to evaluate the role of CDP as a complementary system to help pathologists by the surgeons during frozen section evaluation. In this study, the surgeon followed the standard guideline‐based on frozen pathology, and CDP was applied on IMs after frozen result declaration. The pathologist rechecked his/her negative diagnoses based on CDP‐positive scored samples declared by the surgeons. Combining these studies provides reliable guidance for the surgeon to use CDP intra‐operatively. Pathological classification of IMs, in correlation with the hypoxic metabolisms of breast cells (from atypical hyperplasia to neoplasia^28^, ^29^, ^30^, ^31^, ^32^, ^33^), was clinically investigated to evaluate the main application of CDP as a real‐time diagnostic tool.

## 2 MATERIALS AND METHODS

### 2.1 Study design

Four studies were designed. One for re‐checking the in vivo calibration of CDP in human models and three (named A, B, and C) for evaluating the abilities of CDP as a surgeon assistant. Totally from November 2018 to April 2020, 202 patients were registered and 188 patients with different types of breast tumors (IDC (invasive ductal carcinoma): *n* = 129 (68.6%), ILC (invasive lobular carcinoma): *n* = 6 (3.2%), DCIS (ductal carcinoma in situ): *n* = 35 (18.6%), atypical ductal hyperplasia: *n* = 8 (4.3%), and benign tumors: *n* = 10 (5.3%)) were assigned for CDP clinical studies. Men made up 2 (1%) of the patients; 186 (99%) were women, and all were from the White/Caucasian race. The patients’ age range was 22–76. Among 202 patients, 14 cases were excluded due to the system's noisy responses, refused to participate, or failed pathological specimens in tissue processing procedures. The study is registered at Iran National Committee for Ethics in Biomedical Research (IR.TUMS.VCR.REC.1397.355). Table 1 shows the demographic characteristics of the patients and tumor before the surgery.

**TABLE 1:** Demographic characteristics of the patients and tumor characteristics before surgery

Variable	Patients (*N* = 188)
Age	43.5 (21–71)
Sex	
Female	186 (99%)
Male	2 (1%)
Tumor type	
IDC	135 (72%)
ILC	6 (3%)
DCIS	39 (21%)
Atypical ductal hyperplasia	8 (4%)
Tumor size (min–max)	1.5–8 cm
Tumor location	
Upper outer quadrant (UOQ)	95 (51%)
Upper inner quadrant (UIQ)	23 (12%)
Lower outer quadrant (LOQ)	43 (23%)
Lower inner quadrant (LIQ)	12 (6%)
Nipple and central breast	15 (8%)
Tumor site	
Left breast	130 (69%)
Right breast	58 (31%)
State of patients	
Re‐surgery after re‐currency	8 (4%)
Re‐surgery due to involved margin	5 (3%)
First surgery	175 (93%)

### 2.2 Inclusion criteria

Patients of all ages and genders with breast tumor disease were candidates for breast‐conserving surgery. Presurgical radiological and pathological evaluation results are the main inclusion criteria for using CDP. The cavity side margins of different histologic subtypes of breast tumors such as invasive ductal carcinoma, invasive lobular carcinoma, and malignant phyllodes tumor would be checked by CDP. Cavity side margins must be removed if their intra‐operative tumor side frozen section results were ADH, DCIS, LCIS, IDC, ILC, etc. Breast cancer patients who underwent first‐line breast‐conserving surgery, re‐surgery after re‐currency, or due to involved margin were recruited in this study. The following cases were not important parameters for patient’s recruitments: involved lymph node if she/he does not require chemotherapy before surgery, patient’s age, and surgical history.

### 2.3 Exclusion criteria

Neo‐adjuvant cases with or without obviously remained tumors are excluded from the study.

### 2.4 Proposed protocol for evaluating the reliability of CDP scoring intra‐operatively

To evaluate the reliability of CDP scoring in clinical situations, we applied CDP for detecting both IMs and EMs during breast‐conserving surgery of patients under the Ethics committee confirmation license. Test protocol was approved by the institutional review board of Tehran University of Medical Science (IR.TUMS.VCR.REC.1397.355) with the informed consent of candidate patients. Fourteen of 127 (11%) patients were excluded from the survey, and 113 patients (111 female and 2 male) were included (Figure S1).

As depicted in Movie S1, all of the regions in body side margins named internal margins (IMs) were tested by CDP after tumor dissection. There are six distinguished margins in the tumor side (external margin) with the reciprocal part in body side (internal margin) included: superior, inferior, medial, lateral, superficial, and posterior (deep).^34^

Depending on the size of the tumor and its proximity to one of the margins (not all of the margins), some margins must undergo further analysis. In this regard, the internal regions with more joint boundaries with the tumors would require further scans due to their larger formed internal margins.

The head probe needles are disposable, and the entered length of the needles into the breast cavity side margins is 4 mm, as our surgeons want to be ensured from the absence of any atypical/neoplastic cells or satellite lesions up to the depth of 4 mm in the cavity side after tumor dissection. The sensing head probe contains three needles decorated with multi‐walled carbon nanotubes (MWCNTs) named working, counter, and reference electrodes, with a circular distance of 3 mm in humans and 1 mm in mouse model assays (Figure S2). All were entered into the suspicious tissue for testing (Figure S3).

When the validation of CDP scoring based on pathological categorization was confirmed by in vivo human model study, we started research clinical investigation based on the protocol shown in Movie S1 to minimize the CDP‐based dissected lesion without any perturbation in the standard surgical protocol for patients (frozen and permanent are being performed as a routine procedure for every patient during surgery). Positive margins which must be re‐excised due to frozen section are atypical lesions (FEA, ADH),^35^ LCIS, DCIS, IDC, and ILC. Positive margin in CDP calibration is also similar to the frozen section.^25^ Positive margin in permanent pathology evaluation of tumor which recommends re‐surgery (independent from frozen or CDP) would be started from DCIS while some references also recommend re‐surgery of ADH.^36^

**FIGURE 1:**
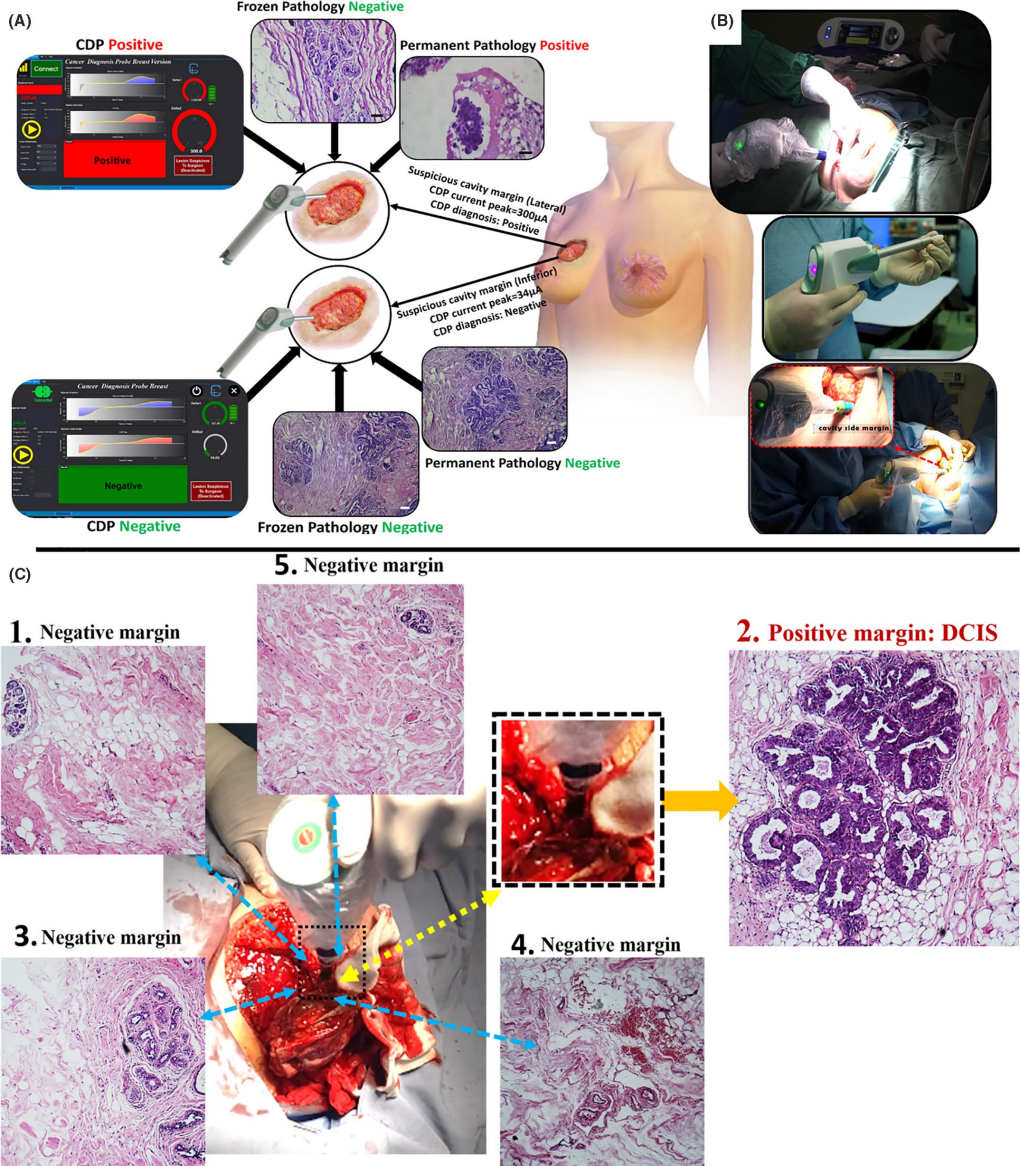
(A) Schematic of applying cancer diagnostic probe (CDP) in real‐time detection of suspicious margins during breast cancer surgery. The assay was conducted on a suspicious margin inside the patient's body (lateral margin of patient ID: 2), which is the significance of CDP. It also positively scored the margin, and the removed specimen showed a negative result for malignancy in frozen analyses. Meanwhile, the permanent H&E showed the papillary lesion with the atypia region, which must be removed by the surgeon. Inferior IM of the other patient (ID 62) was negatively scored by CDP and confirmed by both frozen and permanent H&E as usual hyperplasia and (B) CDP as a surgeon‐assisted tool in the surgery room for finding involved IMs to pre‐invasive/invasive cells. (C) Four neighboring regions of a positive internal margin (had been scored by CDP) were checked by CDP, not only to prevent additional cutting of free lesions but also to remove remained involved regions (Movie S1)

If CDP positively scores a cavity side lesion, its neighbors (with a width of 3 mm) should also be checked by CDP. Totally (as could be observed in Figure S3), a circular region with four quarters should be checked, and CDP would individually test each quarter. If CDP positively scores a quarter, the surgeon should again divide the suspicious region into four assumed sub‐quarters and test each quarter. Finally, a positively scored region with a size of 1 cm^2^ is a candidate for dissection. As a result, the surgeon could excise the involved region with safe neighbors. Moreover, some scattered satellite neoplastic lesions that occurred in some cases (during our investigation) could also be detected by CDP. The entrance of about 4 mm into the tissue depth inside the body would be a good checkpoint (due to the surgeons’ opinion) on the probable presence of neoplastic cells in satellite distribution. In future real clinical use of CDP after passing the standards, the surgeon can dissect the whole of a margin in which even one lesion is positively scored by CDP (similar to frozen protocol). In this trial, all of the margins, which had been scored by CDP and tested by frozen pathology, were individually evaluated by permanent pathology as a gold standard diagnosis based on histological classifications of breast tumors. When a permanent histological pattern was suspicious for pathologists between two different diagnoses (e.g., UDH (usual ductal hyperplasia)) but suspicious to be ADH (atypical ductal hyperplasia), IHC (immunohistochemistry) would be recommended by her/him. Here, we have to make the diagnosis based on the IHC results. For example, patient ID: 93 was diagnosed as sclerosing adenosis by H&E, and the pathologist was not ensured about invasive nature of the cells, but CDP scored it as an active cancer region, and SMMH (smooth muscle myosin heavy chain) confirmed the infiltration of neoplastic cells from the stroma. The schematic of applying CDP as a real‐time tool for the detection of suspicious margins during breast cancer surgery^25^ is presented in Figure 1.

### 2.5 Procedure and methods of clinical studies

#### 2.5.1 Study A

In this observational study, the CDP was started to be used by the surgeon in all of the IMs when the margins were declared free after one or further sequences of frozen evaluation. The frozen section might declare some tumor margins as involved EMs, and through standard guideline, cavity side margins must be re‐excised and resend for frozen up to be declared as free EMs by pathologists. In this clinical study, the surgeon checked and scored the IM lesions by CDP and just recorded the results without informing the pathologist about the positively scored IMs or dissecting the positively scored region to prevent any CDP‐based intervention. In the next step, 2–4 days after, the pathologist would check all of the tumor margins (EMs) through H&E and IHC (if needed). If a margin (which was declared as free margin in frozen) was positive, the frozen missed that margin. If CDP had positively scored the cavity side of that margin, it means that CDP detected the missed margin in frozen. If CDP negatively scored the mentioned margin, it means that CDP missed the margin, similar to frozen. Our gold standard for the margins’ pathological states is the permanent H&E/IHC assay of the tumor side margin.

#### 2.5.2 Study B

In this interventional study registered in IRCT (ID: IRCT20190904044697N1), the surgeon followed the standard guideline for margin re‐excision based on frozen pathology and then immediately applied CDP (just as a complementary diagnostic tool) to check the IMs. In this clinical study, the surgeon just dissects the lesions which were positively scored by CDP. These samples were named “CDP Samples.” On the other hand, the results of frozen pathology on EMs, named as “frozen samples,” were the criterion for the main surgeon to continue and complete the surgery. The required time for checking all of the IMs by CDP was about 10 min (Movie S1). Permanent pathology was carried out on both frozen and CDP samples. Hence, without any sampling bias, the diagnostic role of CDP was evaluated. The gold standard for the margins’ pathological states is permanent pathology of IMs in margins that have CDP samples and permanent pathology of EMs in margins that have not CDP samples. Twenty‐five patients were recruited in this study, and CDP scored 150 margins.

#### 2.5.3 Study C

In this interventional study registered with the ID of IRCT20190904044697N3, when the margins were declared free after one or further sequences of frozen evaluation (frozen might declare some tumor margins as involved EMs and through standard guideline, cavity side margins must be shaved up to be declared as free EMs by pathologists), the CDP was started to be used by the surgeon in all of the IMs. Then, the surgeon checked and scored the IM lesions by CDP and informed the pathologist about the CDP scores. The pathologist further evaluates all over the last reciprocal EMs of positively scored IMs by slide preparation from much more points on that margin (this EM might be the tumor margin or a re‐excised EM). If the pathologist found any suspicious lesions in re‐evaluation, he/she informs the surgeon to remove the positively scored margin, and if not, the surgeon would not remove the CDP‐positive IMs, and we just record the data of CDP responses. In the next days, the pathologist will recheck all of the last reciprocal EMs by permanent H&E. Hence the patient will be recalled to undergo second surgery if any EMs have been missed by frozen and detected in permanent pathology either had been found by CDP or not. Twenty‐five patients were recruited in this study, and 150 margins were evaluated.

### 2.6 Statistical analysis

For the statistical analysis of this study, SPSS software (ver. 26) was used. To evaluate each of the diagnostic tests, the ROC and AUC have been performed to compare detection efficiency between each group based on the permanent pathology as a gold standard. Also, the sensitivity, selectivity, accuracy, and specificity of each study were calculated with SPSS. A *p*‐value lower than 0.01 was considered notable.

## 3 RESULTS

### 3.1 Evaluating the in vivo reliability of CDP scoring versus permanent pathology in BCS cases

**FIGURE 2:**
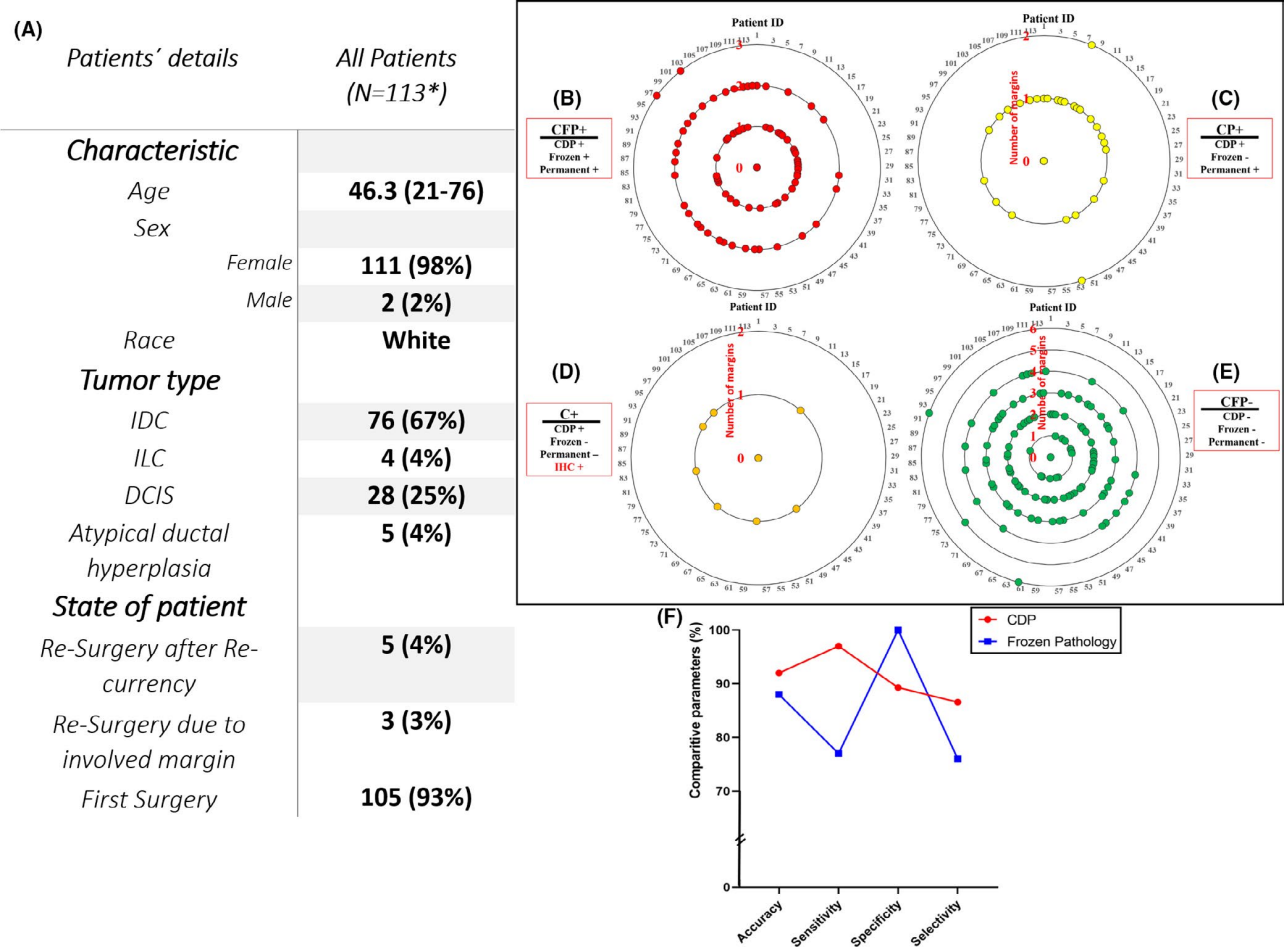
(A) Clinical and pathological characteristics of patients were randomly assigned to this study, investigation of margins in 113 patients with breast cancer during surgery by cancer diagnostic probe (CDP), frozen H&E, permanent H&E, and IHC (if required), (B) The number of patients ID which all three CDP/frozen/permanent was positive (CFP+), (C) The number of patients ID that CDP and permanent was positive and frozen declared negatives (CP+), (D) The number of patient ID which CDP was positive and permanent H&E could not declare final diagnosis. Therefore, IHC was recommended and confirmed CDP results (C+), (E) The number of patients ID which all three CDP/frozen/permanent declared was negative (CFP‐). In each diagram, internal circles indicate the number of tested margins for one patient, (F) Comparison of the accuracy, sensitivity, specificity, and selectivity parameters for CDP, and conventional Frozen pathology for preclinical study. *Among 127 patients, 14 cases were excluded due to noisy responses of the system, refused to participate, and failed pathological specimens in tissue processing procedures

Prior to starting human studies, in vivo checking of the reliability of CDP scoring with respect to permanent pathology was carried out. In this regard, 897 individual EMs and IMs were intraoperatively scored by CDP and diagnosed by pathology under the ethical certificate ID of IR.TUMS.VCR.REC.1397.355 (Figure 2A, Tables S2, S3 for EMs and IMs, respectively).

Seventy‐five of 113 (66%) patients had at least one positively scored IM, reported by CDP and confirmed by the pathological result of permanent or frozen assays (marked as CFP+; Table S3). As an example, a representative CFP+IM with a recorded current peak of 460 µA (patient: ID 18) showed a small lesion of distinguished IDC in the H&E image of the excised sample.

Seventy‐six of 190 (40%) IMs which were positively scored by CDP were diagnosed as cancer‐free lesions after being evaluated by frozen pathology. Hence, these regions were assumed as doubtful samples. Interestingly, permanent H&E sections corroborated the presence of atypical/neoplastic cells in 30/76 of those samples (marked as CP+; Table S3). Anterior IM in patient ID 46 was an example for such samples whose CDP current peak was 247 µA, and the permanent diagnosis was papillary lesion with atypia (positive margin). Among 76 IMs had been positively scored by CDP, 32 IMs were negative in permanent H&E diagnosis, and the CDP scores were rejected (considered as false positive [FP]). Permanent H&E in 14/76 of these margins were suspicious for pathologists, and they could not reach the final diagnosis. Hence, IHC was recommended for them (4 IMs between sclerosing adenosis (SA) and invasive carcinoma, 10 IMs consisted of hyperplastic foci suggestive for being ADH). SA lesions are important patterns that need to be considered because invasive carcinoma might be wrongly missed instead of SA.^38^ Also, it is impossible to perform IHC on frozen samples during surgery. SMMH^39^ and P63^40^ IHC markers were conducted on permanent samples of the suspicious IMs to evaluate if any neoplastic cells infiltrated from the myoepithelial layer. Two of 4 (50%) in those IMs did not express SMMH and P63 IHC markers. Hence, they were diagnosed as invasive carcinoma, and the positive scores of CDP were confirmed. Two other IMs expressed both SMMH and P63, and the positive scores of CDP were rejected (e.g., patients ID 107 & 111; Figure S4A, S4B).

Ten of 14 CP+ samples with suspicious pathology results which had been recommended for IHC, were UDH (lesions such as “moderate DH (Ductal Hyperplasia),” “FCC (Fibrocystic Change) with CCC (Columnar Cell Change),” and “Florid DH”) lesions suggestive of being ADH. Here, cytokeratin (CK) 5/6 and CK14 IHC markers would distinguish these benign lesions from ADH if most suspicious cells were stained with mosaic patterns.^41^ More than one focus of proliferative lesions did not express both CK markers in half of the suspicious hyperplastic samples. Thus, the atypical phenotype of those lesions and positive CDP scores were confirmed (e.g., patient ID 14; Figure S5A & patient ID 96; Figure S5B). Those margins were important distinct diagnoses of CDP (IHC assisted CP+) (Table S3). CK markers were expressed in the other five suspicious hyperplastic samples, and the CDP scores were false (e.g., Patient ID95 superior margin; Figure S5C).

Hence, among 190 IMs that were positively scored by CDP, 151 margins were confirmed through permanent/IHC analyses, while 39 IMs were false positives (FPs) of CDP (Table S4A).

Some clinical naming diagnostic scores had been proposed during the preclinical studies were also used here, such as CFP+ (marked as CDP/frozen/permanent: +/+/+), CP+, C+, and CFP‐. The number of samples scored in each of those categorizations in our clinical study is presented in Figure 2B‐E.

The CDP sensitivity (correct positive scores on involved margins) and accuracy on the total number of IMs and EMs were more than 97% and 92%, respectively (Figure 2F and Tables S5, S6).

False negatives (FNs) margins, which were negatively scored by CDP while were positive under H&E diagnosis, are more important than FPs because the residues of pre‐invasive/invasive lesions in the body might directly induce the disease recurrence probability and reduce the survival rate of the patients. Two regimes of FNs named as high‐value false negatives (HVFNs) and low‐value false negative (LVFN) were observed in CDP scores. HVFNs were the samples that were negatively scored by CDP (in the green or free region of the proposed classification), while permanent H&E diagnosed them as positive margins. These were the samples that must be dissected, but the CDP did not recommend dissection. Just 3 IMs with such scoring were found among 491 in vivo IMs of human samples (e.g., patient ID 105, posterior (deep) margin). A small focus of IDC was observed in frozen and permanent slides of these three margins (marked as false C‐, Figure S6A, and Table S4B).

LVFN samples scored in the yellow region by CDP (They showed current peaks between 150 µA and  203 µA). These margins were diagnosed positive (in the red region) by permanent H&E. Dissection of these lesions would be mandatory, while according to CDP, scoring dissection was not mandatory but might be helpful. Hence, LVFN samples might not be as crucial as HVFN. Four of 163 (2%) positive IMs were falsely scored by CDP in the negative yellow region. Two of 4 (50%) those samples were also falsely diagnosed in frozen sections while one focus of ADH and DCIS was found in their permanent pathology slides (marked as false CF‐) (e.g., Figure S6B and Table S4C). The other two margins were classified as UDH lesions with a small IDC focus in both frozen and permanent assays (marked as false C‐) (Figure S6C and Table S4B). Totally, 7 of 491 (1%) scored IMs by CDP was FN. However, more investigations might be helpful to find other probable FNs of CDP.

It is worth noting that some samples that CDP negatively scored were diagnosed as involved margins to ADH in frozen sections, but permanent pathology confirmed the CDP scoring (e.g., patient ID 94 posterior). These samples were marked as CP‐. Through permanent diagnosis, the negative IMs with matched CDP scores and frozen section diagnoses were marked as CFP‐ samples (e.g., posterior IM of patient ID 46: Table S3).

To prevent sampling bias, CFP‐ samples with lowly suspicious of H&E patterns to abnormal morphologies were re‐checked by further H&E and IHC assays (as had been experimented on FP samples). Results rolled out the presence of any atypical/neoplastic lesions in those samples (e.g., Figure S7).

Very low levels of non‐targeted H_2_O_2_ might be produced in the wounds during surgery,^42^, ^43^ but did not induce any perturbation or false response in CDP scoring as we investigated and discussed in Supplementary (Figure S8).

The CDP sensitivity (correct positive scores on involved margins) and accuracy on the total number of IMs and EMs were more than 97% and 92%, respectively (Section S1).

This precision was achieved in a real‐time manner; meanwhile, the gold standard assay (permanent H&E/IHC) not only requires at least 24 h for sample preparation and staining but also needs an expert pathologist for diagnosis.

The rate of CFP+, CP+, and CFP‐ as correct diagnoses of CDP were 23%, 6%, and 60%, respectively, with 1.4% and 7.9% of false negatives and false positives, respectively.

ROC curve analysis has been performed to compare CDP and frozen conventional pathology with the gold standard (permanent pathology). The result showed that the AUC value for CDP was 0.931 (*p*‐value < 0.0001 and CI99% 0.906–0.955) (Figure S9 and Table S7) in comparison to frozen pathology, 0.881 (*p*‐value < 0.0001 and CI99% 0.844–0.917) (Figure S10 and Table S8). So, CDP has better sensitivity and selectivity (Figure 2F), and it can be used as a diagnostic tool for the detection of preneoplastic/neoplastic cells during surgery. Also, the ROC test result shows that CDP has better results compared to frozen due to the higher area under the curve of CDP (0.931 > 0.881).

Due to this study (Figure 2), the hypoxia approach's efficiency for margin detection in both false negative and positive values was elucidated. To achieve clinical and production certifications for CDP, we designed three clinical studies (one observational and two interventional studies with study registration ID: IRCT20190904044697N1 and IRCT20190904044697N3) (see methods) to present a wide application of this method in helping fast diagnosis of clean and involved margins just by the surgeon through observational and interventional studies. The outcomes of these studies and the system's electrical and safety evaluation exams resulted in the achievement of clinical usage certification for CDP by the Iran Ministry of Health with the national ID number of: 14006918495 and product license number 23212882 as a surgeon assistant tool in breast cancer surgery.

### 3.2 Clinical efficacy of CDP‐based margin detection/cleaning by the surgeon (Studies A, B, and C)

#### 3.2.1 Study A: Observational study

In this study, CDP had no role in margin diagnosis and excision. This study was designed to realize better the impact of CDP in finding involved cavity side margins that may be missed by frozen pathology during breast tumor surgery. In this regard, the surgeon followed the standard guideline based on frozen pathology, and CDP was applied just as a complementary diagnostic tool to check the IMs without any sampling from checked locations. After checking and removing the involved margins through frozen results of EMs, we just recorded the scores of CDP on each internal margin. If just one point in a margin became positive, CDP scored that margin as positive. After receiving the results of permanent pathology on tumor side margins, we compare the CDP score on IMs with frozen of EMs based on permanent results of EMs.

**FIGURE 3:**
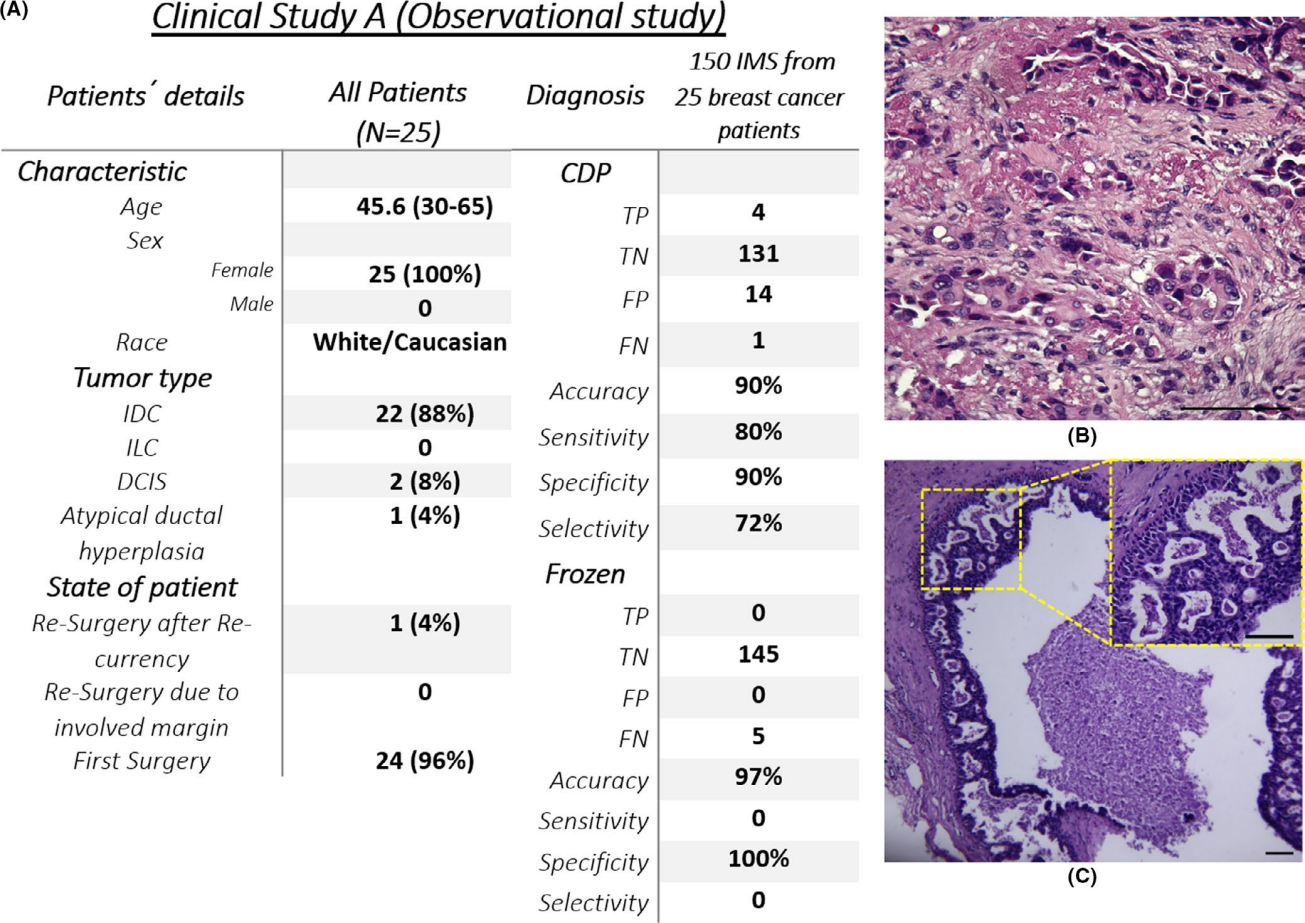
(A) The baseline of the *clinical study A* characteristic and overall study outcome, (B) Cancer diagnostic probe (CDP) positively scored anterior margin of patient ID 114, which was reported as free margin in frozen section but was confirmed as IDC nuclear grade 2 on its reciprocal EMs by permanent pathology, (C) Inferior margin of patient ID 138 positively scored by CDP while frozen declared free margin on its reciprocal margin (EM‐) but permanent pathology diagnosed margin involvement to DCIS on the same EM

Among 25 breast cancer candidates for this study, 4 of 150 (3%) IMs from 4 of 25 (16%) patients which had been positively scored by CDP, were confirmed as involved margins in the permanent evaluation of their reciprocal EMs while they had been reported as free margins in frozen evaluation. Fourteen of 150 (9%) CDP had positively scored IMs, which was not confirmed in the permanent evaluation of their EM reciprocal. One hundred and thirty‐two of 150 (88%) IMs had been negatively scored by CDP (in corroboration to conventional frozen evaluation), while 1 of the reciprocal EMs was declared as involved margin in permanent evaluation (foci of suspicious proliferative UDH with negative CK5/6 and CK14, which was declared as ADH). Totally, in comparison with frozen section (frozen conventional evaluation) as an observational tool, CDP just lost one positive margin while truly scored four missed margins (e.g., Figure 3B anterior margin of patient ID 114; invasive ductal carcinoma nuclear grade 2 and Figure 3C inferior margin of patient ID 138; DCIS). Also, CDP showed 14 overdiagnoses on free margins (Table S9). Also, the sensitivity and specificity of CDP based on permanent evaluation in the first clinical study were evaluated (Figure 3A, Section S2.1., Table S10–S13).

#### 3.2.2 Study B: The independent role of CDP in an interventional study

This study aimed to show the independent role of CDP in margin cleaning in breast‐conserving surgery. So, not only the ability of CDP was evaluated independently, but also the presence of CDP near frozen in helping the patient to have a clear margin was evaluated. Here, both the positively and negatively scored samples by CDP in the patient's IMs were excised and pathologically evaluated. Permanent H&E would evaluate FPs of CDP on samples dissected through CDP scoring, and FNs of CDP might be detected by permanent H&E of EMs if they were positive. Hence, the independent role of CDP in evaluating IMs in comparison with the frozen and permanent evaluation of EMs would be clarified.

**FIGURE 4:**
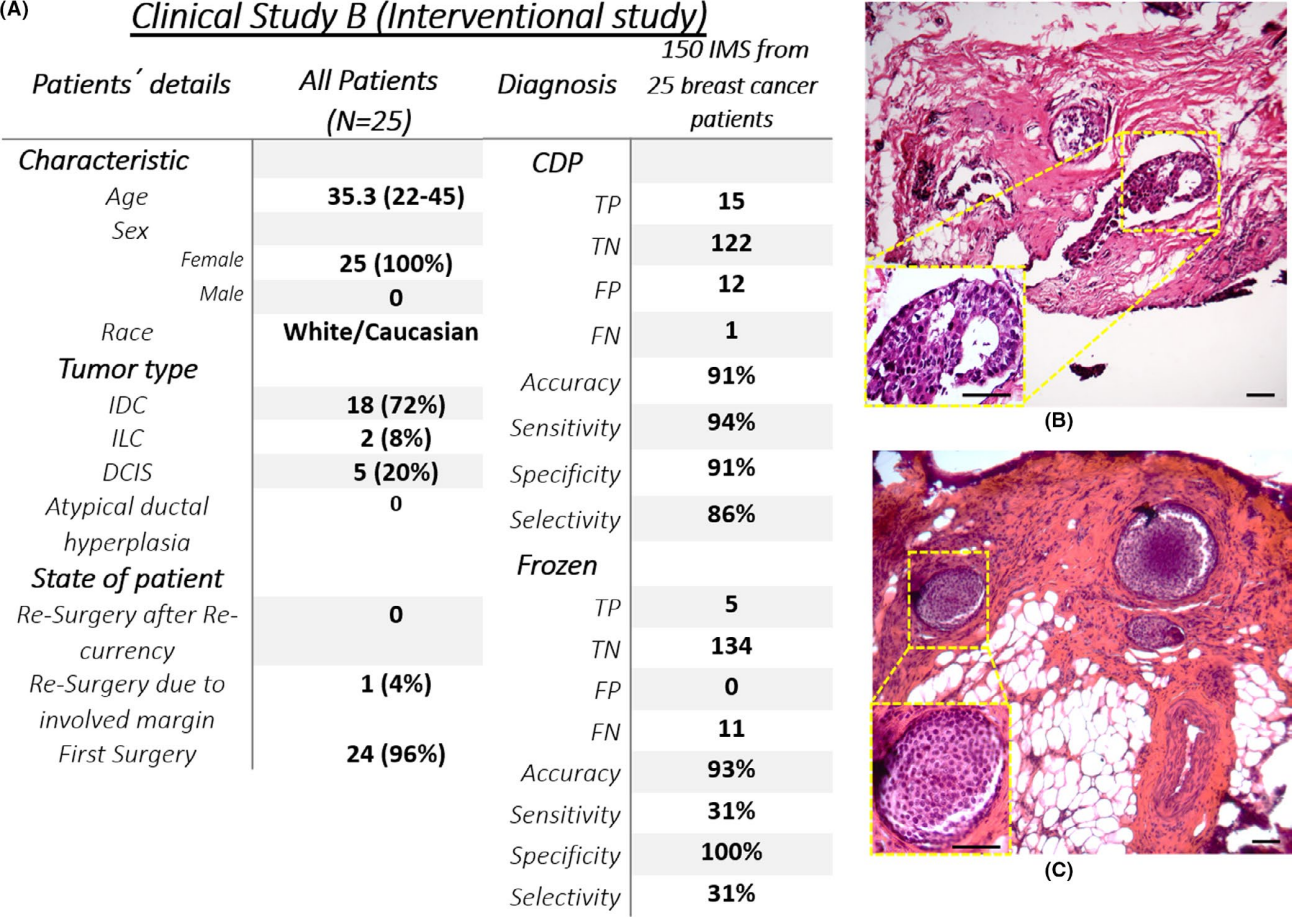
(A) The baseline of the *clinical study B* characteristic and overall study outcome, (B) Invasive ductal carcinoma (IDC) grade 2/DCIS lesions found in an internal margin that positively scored by Cancer diagnostic probe (CDP) while frozen declared free margin on its reciprocal margin (EM‐) but permanent pathology diagnosed margin involvement on the same EM (patient ID:143), (C) LIN2 lesion which CDP score on IM was positive (ID 145), frozen on reciprocal EM was negative, and permanent on reciprocal EM was negative

Eleven of 150 (7%) samples for 8 of 25 (32%) patients recruited in this study were positively scored by CDP and confirmed by permanent H&E of CDP samples, while none of them were diagnosed in frozen sections of their reciprocal EMs (e.g., Figure 4B lateral margin of patient ID 143; IDC grade 2/DCIS). Four of 150 (3%) samples for 2 of 25 (8%) patients were truly scored positive by both CDP and frozen methods. In 122 of 150 (81%) samples, the IMs were negatively scored by CDP, confirmed in permanent H&E of reciprocal EMs. It is worth noting that on one patient (ID 145), no trace of any high‐risk lesion was found neither in frozen nor in permanent of one of the EMs (florid UDH; medial margin) while CDP positively scored its reciprocal IM. Permanent pathological investigations on the scored IM declared the presence of LIN2 (Two foci of LCIS: Figure 4C). However, CDP showed one LVFN (superior margin of patient ID 158), which was correctly diagnosed by frozen analysis (Table S14).

Also, the sensitivity and specificity of CDP and frozen assays in study B were evaluated based on permanent results (Figure 4A, Section S2.2., Table S15–S18).

#### 3.2.3 Study C: The complementary role of CDP in an interventional study

This study was designed to show the role of CDP in helping the pathologist by the surgeon during the surgery and improve the accuracy of the excising specimen from the breast. In this regard, after evaluating the frozen section by the pathologist, the reciprocal IMs of negative EMs were checked by the surgeon with the assistance of CDP, and the pathologist would be informed about the positive IMs with negative EMs. Then pathologist would further evaluate the previous frozen EMs, and if she/he found an involved lesion, the surgeon would be informed and re‐excise the margin. In this regard, the CDP plays a supporting role for the pathologist with the surgeon's assistance. Hence, the negative EMs declared by frozen pathology would be re‐checked by the pathologist if the surgeon informs him/her that CDP positively scored the reciprocal IM.

**FIGURE 5:**
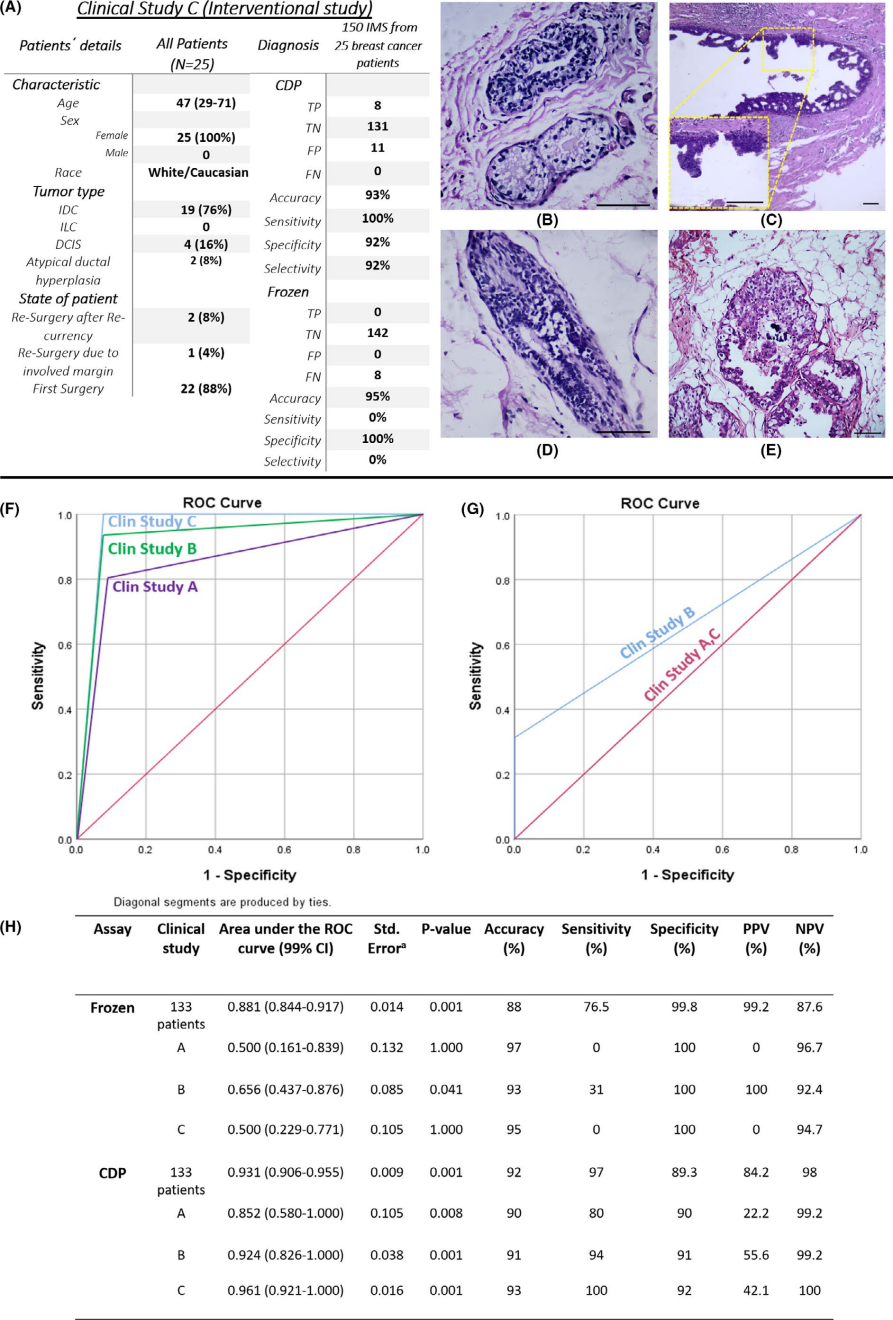
(A) The baseline of the *clinical study C* characteristic and overall study outcome, medial margin of patient ID 183 positively scored by cancer diagnostic probe (CDP) which was reported as, (B) free margin in the frozen section, (C) but was confirmed as a focus of DCIS, intermediate grade on its reciprocal EMs by permanent pathology. (D) Lateral margin of patient ID 187 while frozen declared free margin on its reciprocal margin (EM‐), (E) but permanent pathology diagnosed margin involvement to ADH lesion on the same EM and confirmed CDP, receiver operating characteristic (ROC) diagram for (F) CDP and (G) frozen versus permanent pathology for total 450 EM and IM margins on 75 patients in the three clinical studies. (H) Area under the receiver operating characteristic (ROC) curve, confidence interval, *p*‐value, accuracy, sensitivity, specificity, positive predictive value, and negative predictive value of four clinical study

In this study, among 25 breast cancer candidates for this study, 6 of 150 (4%) IMs from 8 of 25 (32%) patients which had been positively scored by CDP were confirmed as involved margins in the frozen re‐evaluation of their reciprocal EMs while they had been reported as free margins in frozen evaluation. Thirteen of 150 (8.6%) IMs that had been positively scored by CDP were not confirmed in the frozen re‐evaluation of their reciprocal EM. Hence, they were not re‐excised from the surgery site. Permanent pathology not only confirmed all of those 6 of 150 (4%) samples as involved margins but also confirmed the diagnosis of CDP in 2 of 13 (15.4%) EMs had been negatively scored by frozen (3 of 13 (23%) those EMs were declared as suspicious to atypia (ADH) in the permanent evaluation and 1 of 13 (7.7%) was declared as involved to a focus of DCIS, intermediate grade (Figure 5B,C medial margin of Patient ID 183; DCIS and Figure 5D,E lateral margin of patient ID 187; ADH). Two of the suspicious ADH samples were rolled out in CK5/6 and CK14 IHC assays). So, the patients with positive margins were recalled for the second surgery. In the other 11 samples that had been positively scored by CDP and negatively scored by frozen, the CDP score was not confirmed by the reciprocal EM's permanent pathology. Again, the gold standard for the pathological states of both EMs and re‐excised IMs is a permanent H&E/IHC assay (Table S19).

Like previous studies, the sensitivity, and specificity of CDP based on permanent evaluation in this clinical study were evaluated (Figure 5A, Section S2.3., Table S20–S23).

For these clinical studies, ROC and AUC for CDP and frozen conventional pathology were calculated. The area under the curve for CDP in all clinical studies was higher than 0.852 (*p*‐value < 0.008 and CI99% 0.580–1.000), which is higher than this value for frozen conventional pathology (lower than 0.656 [*p*‐value > 0.01 and CI99% 0.437–0.876]). These results for frozen conventional pathology showed that it is not a reliable diagnostic test, and it has not a good balance of sensitivity and specificity (Figure 5F,G).

## 4 DISCUSSION

The main intra‐operative concern in breast‐conserving surgery of the non‐neoadjuvant patients is achieving clear margins, which conventionally could be carried out by a frozen section of tumor margins. However, direct checking of cavity side margins after tumor excision may prevent tumor bed from remaining cancer residues and lead to decreased risk of reoperation and/or healthcare costs. Aside from the limitations of frozen techniques, evaluating just tumor side borders may not be sufficient to be ensured from clearance of cavity side interface,^27^ Hence, a lot of effort has been put into developing new techniques for the direct evaluation of cavity side margins. Cancer diagnostic probe (CDP) showed this ability as a handheld real‐time diagnostic tool with pathological classification. Its mechanism has been based on electrochemical tracing the hypoxia glycolysis, distinctive metabolism of neoplastic cells.^44^, ^45^

Different clinical studies on CDP reported in this investigation were designed to highlight the clinical efficacy of the procedure as a complementary facility near‐frozen section in non‐neoadjuvant BCS cases. Promising results may shed light on using CDP in the absence of frozen in the future. Sensitivity of 93% and specificity of 90% showed the reliable role of CDP as a surgeon assistant in real‐time scanning of IMs based on permanent pathology gold standard of tested lesions.

CDP showed unique impacts as an observational tool which showed great validation with 135 true scores among 150 margins (study A). As an independent diagnostic tool, CDP reduced the number of involved margins that had been missed by frozen pathology (study B). Finally, as a complementary assistant tool, CDP helped the pathologist to re‐check his/her eight miss‐diagnosed EM frozen sections (study C). CDP reduced the number of involved cavity side margins, which had been miss‐diagnosed as clear margins in the frozen section of the tumor side interface. Also, 96% of the involved tumor side margins reported by frozen section had been similarly positively scored as involved cavity side margin by CDP. These results showed that CDP not only approved the true diagnoses of the frozen section about involved margins but also reduced its miss‐diagnoses in 90 margins. Following such trends, applying CDP in BCS surgery not only preserves the role of frozen as the conventional method for intra‐operative margin evaluation but also reduces false negatives that had been missed by the frozen method. Compared with other reported technologies such as MassPen, Margin Probe, and fluorescent biochemical probe,^22^, ^23^, ^46^ CDP has competitive abilities in real‐time diagnosing the involved lesions all over cavity side margins. Also, CDP had been experimented on extensive cohorts of animal and human cases (Table S1). The pathological cut‐off between normal/low‐risk and high‐risk/ neoplastic lesions is considerable due to the metabolism‐based detection mechanism of CDP.

Pathological calibration, real‐time response, easy handling by the surgeon, and metabolism‐based mechanism are the advantages of the CDP. It also can be used as an investigative tool for metabolism‐based research on breast diseases. However, disposable needle shape head probes as a consumption part of the system and customizing the price of disposable head probes are the challenges of CDP, which must be considered. Moreover, the limited number of assayed samples ought to be covered by further studies and trials to better validate the calibration and accuracy of CDP. Also, the ROC test result shows that CDP has better results than frozen due to the higher area under the CDP curve (0.912 vs. 0.828).

Despite promising results, deep investigative analyses must be performed to elaborate the reasons of FPs and FNs for CDP scores. It is worth noting that tested lesions by CDP keep their live dynamic function, and do not need any excision prior to diagnosis. So clear lesions would be conserved in the patient's body (which results in lower mass dissection). Also, positively scored margins can be further evaluated by histological and immune histochemical procedures because the CDP procedure would not destroy the tissue. In contrast to CDP, samples must be dissected, frozen, or fixed to be evaluated by pathological techniques. CDP helps the surgeon for better decision‐making about keeping or excising lesions, especially in retroareolar sites.

Two protocols were suggested to surgeons for the clinical use of CDP due to the results of this paper. First, in the presence of frozen, the CDP could be applied after checking and shaving required margins under the frozen section report of tumor side margins. In this regard, all of the IMs would be re‐checked by CDP. Even the pathologist (who declares the frozen diagnosis) could be informed about the positively scored margins and re‐consider his/her diagnostic decision by further evaluation of the positively scored specimen.

Second, in the centers without the facility of the frozen section, the IMs could be checked by CDP (after tumor dissection) as an independent tool, and positive margins could be re‐excised due to the guidelines described in Figure 1C and Movie S1. Then these margins would be assumed as re‐excised margins for permanent pathological evaluation.

The clinical consequences of applying CDP may be a reduction in the rate of recurrence, minimizing mass dissection from margins which is important in breast conservation and may increase the overall survival of the patients in the future.

## CONFLICT OF INTEREST

Four USA patents (One granted; US Patent US10,786,188 B1, and three publications; US Patent App. US2018/0299401 A1, US2021/0007638 A1, and US2021/0022650 A1) have been published based on this work. M.A. is a member of the scientific advisory board of Arya Nano biosensor Manufacturer Co., a company that is commercializing CDP technology. The remaining authors declare that they have no competing interests.

## AUTHORS’ CONTRIBUTIONS

Zohreh Sadat Miripour: Data curation, Formal analysis, Methodology, and Investigation; Fereshteh Abbasvandi: Validation, Investigation, and Visualization; Parisa Aghaee: Investigation, Formal analysis, and Visualization; Fatemeh Shojaeian: Investigation and Visualization; Mahsa Faramarzpour: Investigation and Visualization; Pooneh Mohaghegh: Investigation and Visualization; Parisa Hoseinpour: Validation, Investigation, and Visualization; Naser Namdar: Software and Methodology; Morteza Hassanpour Amiri: Software and Methodology; Hadi Ghafari: Methodology and Visualization; Sahar NajafiKhoshnoo: Investigation and Visualization; Hassan Sanati: Investigation and Visualization; Mahna Mapar: Investigation and Visualization; Ahmad Kaviani and Nastaran Sadeghian: Validation, Investigation, and Visualization; Mohammad Esmaeil Akbari: Methodology, Validation, and Investigation; Masud Yunesian: Visualization and Validation; Mohammad Abdolahad: Investigation, Conceptualization, Supervision, Project administration, Writing – original draft, and Writing – review & editing.

## ETHICS APPROVAL AND CONSENT TO PARTICIPATE

Patients provided written informed consent according to an ethical approved protocol by the institutional review board of Tehran University of Medical Science (IR.TUMS.VCR.REC.1397.355) at our breast cancer central clinics and assistant hospitals for the use of their samples.

## Supporting information

Supplementary Material

## Data Availability

The authors declare that all the other data supporting the findings of this study are available within the article and its supplementary information files and from the corresponding author upon reasonable request. Also, the authors declare that all codes supporting the findings of this study are available from the corresponding author upon reasonable request.
