# Supplementary material for: Human study on cancer diagnostic probe (CDP) for real‐time excising of breast positive cavity side margins based on tracing hypoxia glycolysis; checking diagnostic accuracy in non‐neoadjuvant cases
Source: Cancer Med. 2022 Feb 28;11(7):1630–45. doi: 10.1002/cam4.4503 (PMC8986141; doi:10.1002/cam4.4503)
Supplement: Supplementary file 1 — Supplementary Material [file CAM4-11-1630-s001.zip › cam44503-sup-0002-Supinfo.pdf]

Clinical Trial Protocol
Iranian Registry of Clinical Trials

20 Feb 2021

In-vivo intra-operative detection of breast margins involved in cancer using hypoxia glycolysis metabolism as neoplastic signaling. A frozen guided clinical trial

Protocol summary

Study aim

Checking the surgical margins in breast cancer patients is a critical step to be ensured from the safe removal of high-risk suspicious cells with minimal dissection of healthy tissues. Remained neoplastic lesions inside the body, induces unavoidable re-surgery and post-surgical therapies which cause many side effects. Here we introduce a real-time intra-operative breast margin checking tool with the capability of diagnosing the pathological state of the tissues.

Design

A pragmatic, cancer patients community-based, single group, randomized trial

Settings and conduct

When frozen declares some tumor margins as involved external margins, through standard guidelines, cavity side margins must be re-excised and resend for frozen up to be reported as free margins. The surgeon will then use CDP to check all the inner margins and inform the pathologist about the positively scored ones. Then, the pathologist further evaluates the last reciprocal external margins of that internal margins by slide preparation from much more points on that margin. If the pathologist found any suspicious lesions in re-evaluation, he/she informs the surgeon to remove the positively scored margin, otherwise, the surgeon wouldn’t remove the CDP positive internal margins, and we record the data of CDP responses.

Participants/Inclusion and exclusion criteria

We select patients from all categories of breast cancer candidates for mastectomy or lumpectomy.

Intervention groups

The surgeon follows the standard guidelines based on frozen pathology. Then CDP will be applied just as a complementary diagnostic tool to check the internal margins after completing the surgery (after checking and removing the involved margins through frozen results of external margins).

Main outcome variables

As a result, without any CDP induced intervention, the impact of CDP in preventing re-surgery would be evaluated.

General information

Reason for update

Acronym

CDP, Cancer Diagnostic Probe

IRCT registration information

IRCT registration number: **IRCT20190904044697N3**
Registration date: **2020-02-23, 1398/12/04**
Registration timing: **registered_while_recruiting**

Last update: **2020-02-23, 1398/12/04**
Update count: **0**

Registration date

2020-02-23, 1398/12/04

Registrant information

Name

Mohammad Abdolahad

Name of organization / entity

Country

Iran (Islamic Republic of)

Phone

+98 21 8802 8367

Email address

m.abdolahad@ut.ac.ir

Recruitment status

Recruitment complete

Funding source

Expected recruitment start date

2020-02-04, 1398/11/15

Expected recruitment end date

2020-03-05, 1398/12/15

Actual recruitment start date
empty

Actual recruitment end date
empty

Trial completion date
empty

Scientific title
In-vivo intra-operative detection of breast margins involved in cancer using hypoxia glycolysis metabolism as neoplastic signaling. A frozen guided clinical trial

Public title
Intra-operative real-time diagnostic system for suspicious breast tissue using electrochemical signaling as a complementary method for frozen section pathology

Purpose
Diagnostic

Inclusion/Exclusion criteria
Inclusion criteria:
Patients with noticeable breast tumor mass or previously diagnosed with solid mass (DCIS, ILC, IDC...).
Exclusion criteria:
No exclusion regarding patients recruitment

Age
No age limit

Gender
Both

Phase
N/A

Groups that have been masked
No information

Sample size
Target sample size: **26**

Randomization (investigator's opinion)
Randomized

Randomization description
Twenty-six patients need to be recruited from all categories of breast cancer for in-vivo tests. Among all types of breast cancers, patients with IDC tumors are more than other types. Our surgical collaborators randomly select and introduce the patients for this trial. Each patient who accepts to take part in the investigation will sign ethical consent.

Blinding (investigator's opinion)
Not blinded

Blinding description

Placebo
Not used

Assignment
Single

Other design features
The system lively determines the H2O2 released from cancer or atypical cells, through reverse Warburg effect and hypoxia assisted glycolysis pathways, in a quantitative electrochemical manner. We proposed a matched clinical diagnostic categorization between the pathological results of the tested tissues and the results of CDP. This categorization is based on ductal intraepithelial neoplasia (DIN) classification (with the latest reported modifications) based on our primary outcome results. Unique ability in the non-invasive and real-time diagnosis of internal margins with pathological values (from high-risk benign to pre-invasive and invasive cancer lesions) makes CDP a distinct intra-operative tool with small and straightforward handheld equipment to increase the prognostic factor of the cancer patients.

Secondary Ids

empty

Ethics committees

1

Ethics committee
Name of ethics committee
Ethics committee of Tehran University of Medical Science
Street address
No.23, 16 Azar Ave, Enghelab Ave.
City
Tehran
Province
Tehran
Postal code
1417863181

Approval date
2018-08-18, 1397/05/27

Ethics committee reference number
IR.TUMS.VCR.REC.1397.355

Health conditions studied

1

Description of health condition studied
Breast cancer surgery

ICD-10 code
C50

ICD-10 code description
Malignant neoplasm of breast

Primary outcomes

1

Description
The pathological classification of different breast lesions correlates with current peaks of CDP. Results show meaningful consistency between DIN (Ductal Intraepithelial Neoplasia) based pathological diagnosis and CDP scoring.

Timepoint
Results will be compared with permanent pathology 3 days after the intervention.

Method of measurement
Electrochemical Cyclic voltammetry

Secondary outcomes

empty

Intervention groups

1

Description

Intervention group: The surgeon follows the standard guidelines based on frozen pathology, and will apply CDP as a complementary diagnostic tool to check the inner margins after completing the surgery (after checking and removing the involved margins through frozen results of external margins). Hence, when the margins are declared free after one or further sequences of frozen evaluation, the surgeon will use CDP on all internal margins. Frozen may report some tumor margins as involved external margins, which through standard guidelines, cavity side margins must be re-excised and resend for frozen up to be declared as free external margins by pathologists. In this clinical trial, the surgeon checks the internal lesions by CDP and inform the pathologist about the positively scored ones. Then, the pathologist further evaluates all over the last reciprocal external margin of that inner one by slide preparation from much more points on that margin. If the pathologist found any suspicious lesions in re-evaluation, he/she informs the surgeon to remove the positively scored margin. Otherwise, the surgeon wouldn't remove the CDP's positive reported margins, and we record the data of CDP responses. In the next step, the pathologist will recheck all of the last reciprocal external margins by permanent H&E. Hence, the patient will be recalled to undergone second surgery if any external margins have been missed by frozen and detected in permanent pathology either CDP has detected them or not.

Category

Diagnosis

Recruitment centers

1

Recruitment center

Name of recruitment center

Noor afshar hospital

Full name of responsible person

Dr Mohammad Abdolahad

Street address

Sadeghin Alley (17th West), Khodaverdi St, Pourebtehaj (Kashanak) St, Bahonar (Niavaran) Sq, Tehran, Iran

City

Tehran

Province

Tehran

Postal code

1978734763

Phone

+98 21 2282 4069

Fax

+98 21 2282 4060

Email

m.abdolahad@ut.ac.ir

Web page address

https://noor-afshar.ir

2

Recruitment center

Name of recruitment center

Khatam Ol Anbia hospital

Full name of responsible person

Dr Mohammad Abdolahad

Street address

Vali Asr Ave, Rashid Yasemi Street, Tehran, Iran

City

Tehran

Province

Tehran

Postal code

1996835911

Phone

+98 21 8888 4040

Email

m.abdolahad@ut.ac.ir

Web page address

https://www.khatamhospital.org

3

Recruitment center

Name of recruitment center

Motamed cancer institute, Breast cancer research center

Full name of responsible person

Dr Mohammad Abdolahad

Street address

No. 45, Shahid Nazari Avenue, Aboureihan street, Enghelab square, Tehran, Iran

City

Tehran

Province

Tehran

Postal code

1315685981

Phone

+98 21 8887 6869

Email

Info@ibcrc.ir

Web page address

http://ibcrc.ir

Sponsors / Funding sources

1

Sponsor

Name of organization / entity

Iran Nano Fund

Full name of responsible person

Dr Mohammad Hosein Bahreini

Street address

No. 38, West Nastaran Ave (Arab), Khorramshahr St., North Sohrevardi St., Tehran

City
Tehran

Province
Tehran

Postal code
1533984611

Phone
+98 21 8876 9188

Email
info@nanofund.ir

Web page address
http://nanofund.ir

Grant name

Grant code / Reference number

Is the source of funding the same sponsor organization/entity?
Yes

Title of funding source
Iran Nano Fund

Proportion provided by this source
70

Public or private sector
Private

Domestic or foreign origin
Domestic

Category of foreign source of funding
empty

Country of origin

Type of organization providing the funding
Industry

Person responsible for general inquiries

Contact

Name of organization / entity
Tehran University of Medical Sciences

Full name of responsible person
Mohammad Abdolahad

Position
Associate professor

Latest degree
Ph.D.

Other areas of specialty/work
Breast cancer surgery

Street address
Nano bio electronic lab, ground floor, school of electrical and computer engineering, university of Tehran faculty of engineering, North Kargar Ave.

City
Tehran

Province
Tehran

Postal code
1439957131

Phone
+98 21 8802 8367

Email
m.abdolahad@ut.ac.ir

Web page address
https://nbel.ut.ac.ir

Person responsible for scientific inquiries

Contact

Name of organization / entity
Tehran University of Medical Sciences

Full name of responsible person
Mohammad Abdolahad

Position
Associate professor

Latest degree
Ph.D.

Other areas of specialty/work
Breast cancer surgery

Street address
Nano bio electronic lab, ground floor, school of electrical and computer engineering, university of Tehran faculty of engineering, North Kargar Ave.

City
Tehran

Province
Tehran

Postal code
1439957131

Phone
+98 21 8802 8367

Fax

Email
m.abdolahad@ut.ac.ir

Web page address
http://nbel.ut.ac.ir

Person responsible for updating data

Contact

Name of organization / entity
Tehran University of Medical Sciences

Full name of responsible person
Mohammad Abdolahad

Position
Associate professor

Latest degree
Ph.D.

Other areas of specialty/work
Breast cancer surgery

Street address
Nano bio electronic lab, ground floor, school of electrical and computer engineering, university of Tehran faculty of engineering, North Kargar Ave.

City
تهران

Province
Tehran

Postal code
1439957131

Phone
+98 21 8802 8367

Fax

Email
m.abdolahad@ut.ac.ir

Web page address
http://nbel.ut.ac.ir

Sharing plan

Deidentified Individual Participant Data Set (IPD)
Yes - There is a plan to make this available
Study Protocol
Yes - There is a plan to make this available
Statistical Analysis Plan
Not applicable
Informed Consent Form
Yes - There is a plan to make this available
Clinical Study Report
Yes - There is a plan to make this available
Analytic Code
Not applicable
Data Dictionary
Not applicable
Title and more details about the data/document
80% of the information about the primary outcome after de-identifying the participants, can be shared.
When the data will become available and for how long
Starting six months after publication.
To whom data/document is available
The data will be available for medical staff and academic institutions.
Under which criteria data/document could be used
Non-identifiable personal data will not be usable for the applicant and will only inform the patient of a positive clinical trial that the device is functioning properly.
From where data/document is obtainable
Applicants can request data access via email. The data will be available to the applicant in a categorized manner with pathology reports.
What processes are involved for a request to access data/document
We will send the data to the applicant in one week.
Comments
