# Supplementary material for: Human study on cancer diagnostic probe (CDP) for real‐time excising of breast positive cavity side margins based on tracing hypoxia glycolysis; checking diagnostic accuracy in non‐neoadjuvant cases
Source: Cancer Med. 2022 Feb 28;11(7):1630–45. doi: 10.1002/cam4.4503 (PMC8986141; doi:10.1002/cam4.4503)
Supplement: Supplementary file 1 — Supplementary Material [file CAM4-11-1630-s001.zip › cam44503-sup-0004-Supinfo.pdf]

Supplementary

Human study on cancer diagnostic probe (CDP) for real-time excising of breast positive cavity side margins in non-neoadjuvant patients; diagnostic accuracy

Table Sup1. Comparative capabilities of various margin detection technologies

Technology	Tested Margin numbers/patients	Human in vivo/In vitro tests	Type of cancer tested	Declared Sensitivity / specificity	Diagnosis declaration time	The cost of the test	The cost of the device
**Mass Spec Pen** [1], [2]	100 patients	In vitro and in vivo on the tumor side margins	healthy and diseased thyroid, parathyroid, lymph node, breast, pancreatic and bile duct tissues	96.4% / 96.2%	10 sec.	100$	100000$
**Margin probe** [3]	76 specimens and 753 points	In vitro on the tumor side margins	Breast	70% / 70%	1 to 5 sec.	each probe €600 per operation	MarginProbe console costs approximately €28,000
**Confocal endomicroscopy (CONVIVO)** [4]	74 patients	In vitro and in vivo on the tumor side margins	Brain	Meningioma: 97% / 91% Glioma: 91% / 94%	The mean imaging time was 5.8 minutes per patient (range 1.4–17.0 minutes)	–	510000$
**CDP**	1500 human in-vivo clinical breast samples from 188 patients	In vitro and in vivo on the cavity side margins	Breast	93% / 90%	15 sec.	each head probe 5$	15000$

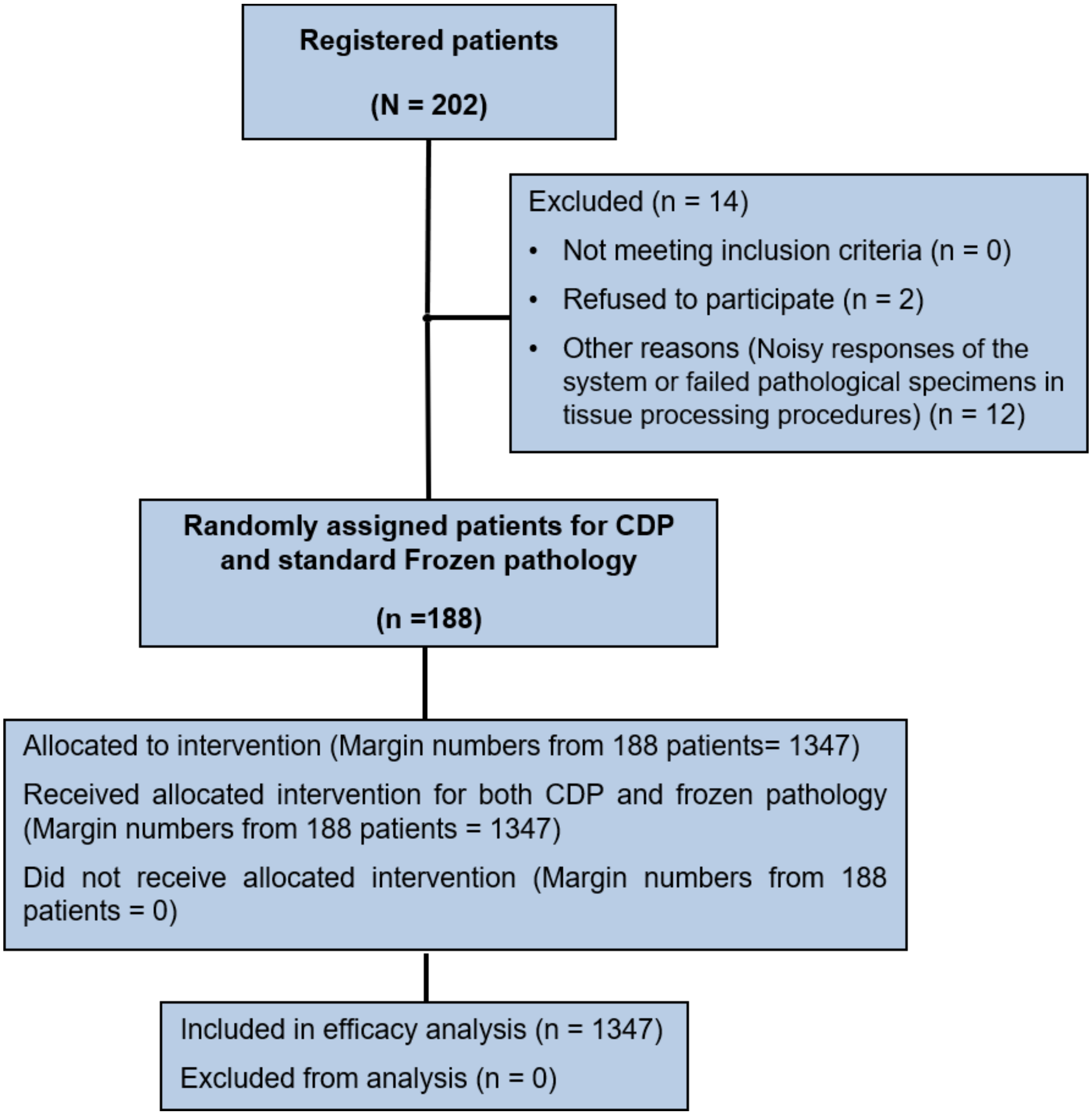

Figure sup1. CONSORT diagram for CDP and standard Frozen pathology in the efficacy analysis.

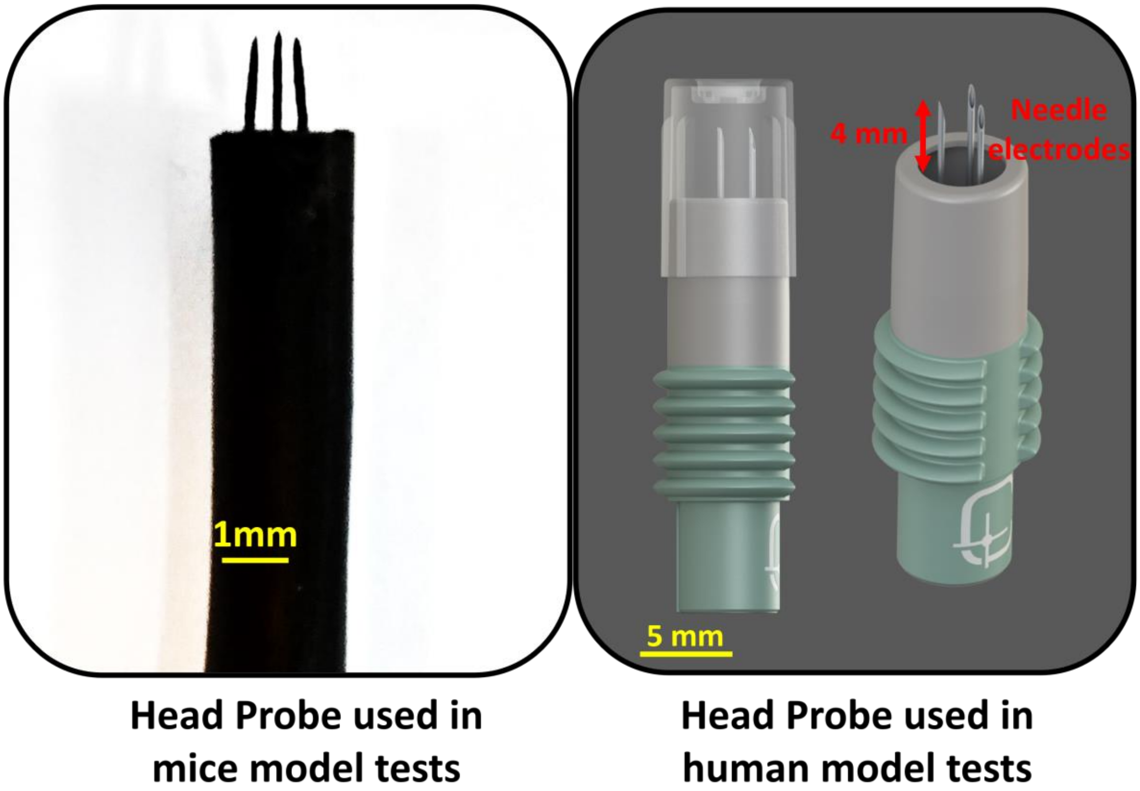

Figure sup2. CDP head probes used for a) mice and b) human model tests

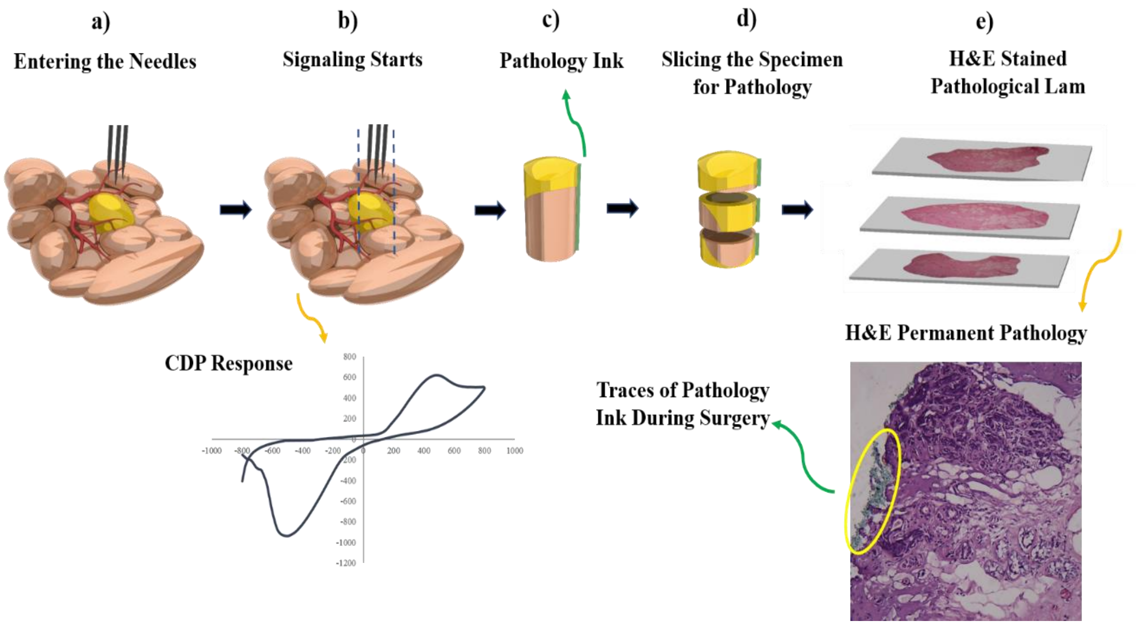

Figure sup3. Schematic of CDP testing procedure includes, a) entrance of the needles to the tissue, b) signal recording from the lesion and cutting the lesion in a cylindrical shape, c) marking the boundary of the lesion by pathology ink, d) slicing the lesion for pathological staining and d) preparing the stained slides through H&E protocol.

Table Sup2. Comparative diagnostic results between CDP and pathological assays on EMs of resected breast tumors in 113 patients based on permanent (Per) gold standard. N.S.M refers to a non-scored margin.

	Gender	Margins of resected Breast tumors					
Patient ID	Female(F) /Male(M)	Anterior CDP/ Fro* /Per*	Posterior CDP/ Fro /Per	Inferior CDP/ Fro /Per	Superior CDP/ Fro /Per	Lateral CDP/ Fro /Per	Medial CDP/ Fro /Per
1	F	+/ +/ +	N.S.M*	-/ -/ -	+/ -/ +	-/ -/ -	+/ +/ +
2	F	N.S.M	-/ -/ -	N.S.M	-/ -/ -	+/ -/ +	-/ -/ -
3	F	N.S.M	N.S.M	N.S.M	N.S.M	N.S.M	N.S.M
4	F	-/ -/ -	+/ +/ +	N.S.M	N.S.M	N.S.M	+/-/- (Second H&E -)
5	F	N.S.M	N.S.M	+/ -/ +	+/ +/ +	-/ -/ -	N.S.M
6	F	N.S.M	N.S.M	N.S.M	N.S.M	N.S.M	N.S.M
7	F	N.S.M	-/ -/ -	N.S.M	+/ +/ +	+/ -/ +	N.S.M
8	F	+/ +/ +	N.S.M	+/ +/ +	+/ -/ +	N.S.M	+/ -/ +
9	F	N.S.M	N.S.M	N.S.M	N.S.M	N.S.M	-/ -/ -
10	F	+/-/- (Second H&E -)	-/ -/ -	+/ -/ +	N.S.M	N.S.M	N.S.M
11	F	+/ +/ +	N.S.M	-/ -/ -	N.S.M	+/ -/ +	-/ -/ -
12	F	+/ +/ +	N.S.M	N.S.M	-/ -/ -	+/ -/ +	N.S.M
13	F	N.S.M	N.S.M	+/ +/ +	N.S.M	-/ -/ -	+/ +/ +
14	F	N.S.M	+/ +/ +	N.S.M	+/ +/ +	N.S.M	+/ -/ - (**IHC +)
15	F	N.S.M	N.S.M	N.S.M	N.S.M	N.S.M	N.S.M
16	F	N.S.M	N.S.M	N.S.M	+/ -/ +	N.S.M	+/ +/ +
17	F	-/ -/ -	-/ -/ -	N.S.M	N.S.M	N.S.M	-/ -/ -
18	F	+/ +/ +	-/ -/ -	N.S.M	N.S.M	+/ +/ +	+/ +/ +
19	F	N.S.M	N.S.M	-/ -/ -	+/ -/ +	N.S.M	+/-/-
20	F	N.S.M	N.S.M	N.S.M	-/ -/ -	N.S.M	-/ -/ -
21	F	N.S.M	+/ +/ +	-/ -/ -	+/-/- (Second H&E -)	+/ +/ +	N.S.M
22	F	+/ +/ +	N.S.M	+/ +/ +	+/ -/ +	N.S.M	-/ -/ -
23	F	N.S.M	+/ +/ +	N.S.M	N.S.M	N.S.M	N.S.M
24	F	-/ -/ -	N.S.M	+/ -/ +	-/ -/ -	N.S.M	N.S.M
25	M	+/-/- (Second H&E -)	-/ -/ -	-/ -/ -	N.S.M	-/ -/ -	N.S.M

**26**	F	N.S.M	N.S.M	N.S.M	+/ +/ +	-/ -/ -	+/ -/ +
**27**	F	+/ +/ +	N.S.M	+/ +/ +	N.S.M	-/ -/ -	N.S.M
**28**	F	+/ +/ +	N.S.M	N.S.M	-/ -/ -	+/ +/ +	-/ -/ -
**29**	F	N.S.M	+/ +/ +	N.S.M	N.S.M	N.S.M	+/ +/ +
**30**	F	-/ -/ -	N.S.M	N.S.M	N.S.M	-/ -/ -	+/ +/ +
**31**	F	+/ +/ +	N.S.M	N.S.M	+/ +/ +	N.S.M	-/ -/ -
**32**	F	**+/-/-**	-/ -/ -	+/ +/ +	N.S.M	N.S.M	+/ +/ +
**33**	F	-/ -/ -	+/ +/ +	-/ -/ -	+/ +/ +	-/ -/ -	N.S.M
**34**	F	N.S.M	+/ +/ +	+/ +/ +	+/ +/ +	N.S.M	N.S.M
**35**	F	N.S.M	**+/-/- (Second H&E -)**	-/ -/ -	N.S.M	-/ -/ -	+/ -/ +
**36**	F	N.S.M	N.S.M	+/ +/ +	-/ -/ -	N.S.M	-/ -/ -
**37**	F	-/ -/ -	N.S.M	N.S.M	-/ -/ -	+/ +/ +	N.S.M
**38**	F	N.S.M	+/ +/ +	N.S.M	N.S.M	N.S.M	N.S.M
**39**	F	-/ -/ -	-/ -/ -	N.S.M	-/ -/ -	-/ -/ -	+/ +/ +
**40**	F	-/ -/ -	+/ +/ +	N.S.M	N.S.M	-/ -/ -	N.S.M
**41**	F	N.S.M	N.S.M	+/ -/ +	N.S.M	-/ -/ -	N.S.M
**42**	F	-/ -/ -	N.S.M	N.S.M	N.S.M	N.S.M	-/ -/ -
**43**	F	N.S.M	+/ +/ +	-/ -/ -	-/ -/ -	+/ +/ +	N.S.M
**44**	F	N.S.M	N.S.M	+/ +/ +	N.S.M	-/ -/ -	N.S.M
**45**	F	N.S.M	N.S.M	-/ -/ -	-/ -/ -	**+/-/-**	**+/-/- (Second H&E -)**
**46**	F	N.S.M	-/ -/ -	**+/ -/ Suspicious (IHC +)**	N.S.M	N.S.M	N.S.M
**47**	F	N.S.M	N.S.M	-/ -/ -	-/ -/ -	+/ +/ +	+/ +/ +
**48**	F	+/ +/ +	N.S.M	N.S.M	N.S.M	-/ -/ -	+/ -/ +
**49**	F	-/ -/ -	N.S.M	N.S.M	+/ +/ +	N.S.M	-/ -/ -
**50**	F	N.S.M	-/ -/ -	-/ -/ -	N.S.M	-/ -/ -	N.S.M
**51**	F	-/ -/ -	N.S.M	N.S.M	N.S.M	N.S.M	+/ -/ +
**52**	F	N.S.M	N.S.M	N.S.M	N.S.M	+/ -/ +	+/ -/ +
**53**	F	**+/-/- (Second H&E -)**	+/ +/ +	+/ +/ +	-/ -/ -	-/ -/ -	N.S.M
**54**	F	+/ +/ +	N.S.M	-/ -/ -	N.S.M	N.S.M	N.S.M
**55**	F	+/ +/ +	N.S.M	N.S.M	-/ -/ -	-/ -/ -	N.S.M
**56**	F	N.S.M	N.S.M	N.S.M	+/ +/ +	N.S.M	-/ -/ -
**57**	F	+/ +/ +	-/ -/ -	N.S.M	-/ -/ -	N.S.M	+/ +/ +
**58**	F	N.S.M	N.S.M	+/ +/ +	**+/ -/ - (IHC+)**	+/ +/ +	N.S.M
**59**	F	N.S.M	N.S.M	-/ -/ -	N.S.M	N.S.M	-/ -/ -
**60**	F	-/ -/ -	+/ +/ +	+/ +/ +	+/ +/ +	N.S.M	N.S.M
**61**	F	N.S.M	-/ -/ -	N.S.M	N.S.M	+/ +/ +	N.S.M

**62**	F	N.S.M	N.S.M	-/ -/ -	-/ -/ -	N.S.M	-/ -/ -
**63**	F	N.S.M	+/ +/ +	-/ -/ -	N.S.M	+/ +/ +	+/ +/ +
**64**	F	+/ +/ +	-/ -/ -	+/ +/ +	-/ -/ -	**+/-/- (Second H&E -)**	N.S.M
**65**	F	-/ -/ -	N.S.M	N.S.M	+/ +/ +	+/ +/ +	-/ -/ -
**66**	F	N.S.M	+/ +/ +	+/ +/ +	-/ -/ -	N.S.M	+/ +/ +
**67**	F	**+/-/- (Second H&E -)**	-/ -/ -	+/ -/ +	+/ +/ +	N.S.M	-/ -/ -
**68**	M	-/ -/ -	-/ -/ -	N.S.M	N.S.M	-/ -/ -	-/ -/ -
**69**	F	+/ +/ +	N.S.M	-/ -/ -	+/ +/ +	-/ -/ -	N.S.M
**70**	F	N.S.M	+/ +/ +	N.S.M	+/ -/ - (IHC+)	-/ -/ -	N.S.M
**71**	F	N.S.M	**+/-/- (IHC -)**	+/ +/ +	-/ -/ -	+/ +/ +	-/ -/ -
**72**	F	-/ -/ -	N.S.M	N.S.M	+/ +/ +	N.S.M	+/ +/ +
**73**	F	N.S.M	+/ +/ +	-/ -/ -	-/ -/ -	+/ -/ +	-/ -/ -
**74**	F	N.S.M	-/ -/ -	N.S.M	N.S.M	N.S.M	N.S.M
**75**	F	+/ +/ +	N.S.M	N.S.M	-/ -/ -	+/ +/ +	N.S.M
**76**	F	-/ -/ -	N.S.M	N.S.M	N.S.M	N.S.M	-/ -/ -
**77**	F	N.S.M	+/ +/ +	+/ +/ +	-/ -/ -	+/ +/ +	N.S.M
**78**	F	N.S.M	-/ -/ -	-/ -/ -	N.S.M	-/ -/ -	N.S.M
**79**	F	N.S.M	**+/-/-**	-/ -/ -	-/ -/ -	+/ +/ +	N.S.M
**80**	F	-/ -/ -	N.S.M	-/ -/ -	N.S.M	+/ -/ +	+/ +/ +
**81**	F	-/ -/ -	N.S.M	+/ +/ +	N.S.M	-/ -/ -	-/ -/ -
**82**	F	+/ +/ +	N.S.M	N.S.M	+/ +/ +	N.S.M	+/ -/ - (IHC+)
**83**	F	N.S.M	N.S.M	N.S.M	N.S.M	N.S.M	-/ -/ -
**84**	F	-/ -/ -	+/ +/ +	N.S.M	-/ -/ -	N.S.M	+/ +/ +
**85**	F	N.S.M	N.S.M	-/ -/ -	N.S.M	N.S.M	N.S.M
**86**	F	N.S.M	N.S.M	-/ -/ -	N.S.M	-/ -/ -	-/ -/ -
**87**	F	-/ -/ -	N.S.M	N.S.M	-/ -/ -	+/ +/ +	+/ +/ +
**88**	F	N.S.M	-/ -/ -	N.S.M	N.S.M	N.S.M	N.S.M
**89**	F	N.S.M	+/ +/ +	+/ +/ +	N.S.M	N.S.M	-/ -/ -
**90**	F	N.S.M	-/ -/ -	-/ -/ -	N.S.M	-/ -/ -	+/ +/ +
**91**	F	**+/-/- (Second H&E -)**	N.S.M	-/ -/ -	+/ +/ +	+/ +/ +	N.S.M
**92**	F	N.S.M	+/ +/ +	N.S.M	+/ -/ +	N.S.M	N.S.M
**93**	F	N.S.M	N.S.M	N.S.M	-/ -/ -	N.S.M	-/ -/ -
**94**	F	-/ -/ -	-/ -/ -	+/ -/ +	+/ +/ +	-/ -/ -	-/ -/ -
**95**	F	+/ +/ +	+/ +/ +	-/-/-	**+/-/- (IHC -)**	-/ -/ -	-/ -/ -
**96**	F	+/ +/ +	-/ -/ -	+/ +/ +	+/ -/ +	-/ -/ -	-/ -/ -
**97**	F	-/ -/ -	-/ -/ -	+/ +/ +	+/ +/ +	-/ -/ -	+/ -/ +

**98**	F	+/ +/ +	-/ -/ -	-/ -/ -	-/ -/ -	+/ -/ Suspicious (IHC +)	-/ -/ -
**99**	F	+/ +/ +	-/ -/ -	-/ -/ -	+/ +/ +	+/ +/ +	+/ -/ Suspicious (IHC +)
**100**	F	-/ -/ -	+/ +/ +	+/ +/ +	-/ -/ -	-/ -/ -	+/ +/ +
**101**	F	+/ +/ +	-/ -/ -	+/ +/ +	-/ +/ +	-/ -/ -	-/ -/ -
**102**	F	+/ -/ Suspicious (IHC +)	-/ -/ -	+/-/- (Second H&E -)	+/ +/ +	+/ +/ +	-/ -/ -
**103**	F	-/-/-	-/-/-	+/-/+	-/-/-	+/+/+	+/+/+
**104**	F	-/-/-	-/ +/ +	+/-/+	-/-/-	-/-/-	+/-/- (IHC -)
**105**	F	+/+/+	-/-/-	-/-/-	-/-/-	-/-/-	-/-/-
**106**	F	-/-/-	-/ +/ +	+/+/+	-/-/-	+/+/+	-/-/-
**107**	F	-/-/-	+/ +/ +	-/-/-	+/-/+	+/+/+	+/+/+
**108**	F	+/ -/ Suspicious (IHC +)	+/ +/ +	+/ +/ +	-/-/-	-/-/-	-/-/-
**109**	F	-/-/-	+/ -/ Suspicious (IHC -)	-/-/-	-/-/-	-/-/-	-/-/-
**110**	F	-/-/-	-/-/-	-/-/-	-/-/-	-/-/-	+/-/-
**111**	F	+/ +/ +	-/-/-	+/ +/ +	+/ +/ +	-/-/-	-/-/-
**112**	F	-/-/-	+/-/- (Second H&E -)	+/ -/ Suspicious (IHC +)	-/-/-	-/-/-	-/-/-
**113**	F	-/-/-	+/ +/ +	-/-/-	+/ +/ +	-/-/-	-/-/-

**Red**	Positive margins detected by CDP and confirmed by permanent /IHC
**Green**	Negative margins detected by CDP and confirmed by permanent/IHC
**Highlighted blue**	False negatives of CDP
**Highlighted yellow**	False positives of CDP
**N.S.M***	Non-scored margin
**Fro*/Per***	Frozen Pathology/ Permanent Pathology and IHC

Table Sup3. Comparative diagnostic results between CDP and pathological assays on IMs of resected breast tumors in 113 patients based on permanent (Per) gold standard. The accuracies of CDP and frozen were compared based on permanent/IHC results. N.S.M. refers to a non-scored margin.

	Gender	Margins of Breast tissue (CDP test Inside the Body)					
Patient ID	Female(F) /male(M)	Anterior CDP/ Fro* /Per*	Posterior CDP/ Fro /Per	Inferior CDP/ Fro /Per	Superior CDP/ Fro /Per	Lateral CDP/ Fro /Per	Medial CDP/ Fro /Per
**1**	F	+/ +/ +	N.S.M*	+/-/- (Second H&E -)	+/ –/ +	-/ -/ -	+/ +/ +
**2**	F	N.S.M	N.S.M	N.S.M	-/ -/ -	+/ –/ +	-/ -/ -

**3**	F	-/ -/ -	-/ -/ -	N.S.M	+/ +/ +	N.S.M	+/ +/ +
**4**	F	-/ -/ -	N.S.M	-/ -/ -	-/ -/ -	**+/-/- (Second H&E -)**	N.S.M
**5**	F	-/ -/ -	N.S.M	+/ –/ +	+/ +/ +	N.S.M	N.S.M
**6**	F	N.S.M	N.S.M	-/ -/ -	N.S.M	-/ -/ -	-/ -/ -
**7**	F	N.S.M	**+/-/- (IHC -)**	-/ -/ -	+/ +/ +	+/ –/ +	-/ -/ -
**8**	F	+/ +/ +	N.S.M	+/ +/ +	+/ –/ +	N.S.M	+/ –/ +
**9**	F	N.S.M	N.S.M	N.S.M	-/ -/ -	-/ -/ -	-/ -/ -
**10**	F	-/ -/ - **(Second H&E -)**	-/ -/ -	+/ –/ +	-/ -/ -	-/ -/ -	N.S.M
**11**	F	+/ +/ +	**+/-/- (Second H&E -)**	-/ -/ -	N.S.M	+/ –/ +	N.S.M
**12**	F	+/ +/ +	-/ -/ -	-/ -/ -	-/ -/ -	+/ –/ +	N.S.M
**13**	F	N.S.M	-/ -/ -	N.S.M	-/ -/ -	-/ -/ -	+/ +/ +
**14**	F	-/ -/ -	+/ +/ +	N.S.M	+/ +/ +	N.S.M	+/ –/ – **(**IHC +)**
**15**	F	-/ -/ -	N.S.M	-/ -/ -	N.S.M	**+/-/- (IHC -)**	N.S.M
**16**	F	N.S.M	N.S.M	-/ -/ -	+/ –/ +	-/ -/ -	+/ +/ +
**17**	F	-/ -/ -	-/ -/ -	N.S.M	N.S.M	N.S.M	-/ -/ -
**18**	F	+/ +/ +	-/ -/ -	N.S.M	-/ -/ -	N.S.M	+/ +/ +
**19**	F	-/ -/ -	N.S.M	-/ -/ -	+/ –/ +	-/ -/ -	-/ -/ -
**20**	F	N.S.M	N.S.M	**+/-/- (Second H&E -)**	-/ -/ -	-/ -/ -	-/ -/ - **(Second H&E -)**
**21**	F	N.S.M	-/ -/ -	-/ -/ -	-/ -/ -	+/ +/ +	N.S.M
**22**	F	+/ +/ +	N.S.M	N.S.M	+/ –/ +	N.S.M	-/ -/ -
**23**	F	-/ -/ -	+/ +/ +	N.S.M	N.S.M	N.S.M	-/ -/ -
**24**	F	**+/-/- (Second H&E -)**	N.S.M	+/ –/ +	-/ -/ -	N.S.M	N.S.M
**25**	M	-/ -/ -	N.S.M	-/ -/ -	N.S.M	-/ -/ -	N.S.M
**26**	F	N.S.M	-/ -/ -	N.S.M	+/ +/ +	N.S.M	+/ –/ +
**27**	F	+/ +/ +	-/ -/ -	N.S.M	N.S.M	-/ -/ -	N.S.M
**28**	F	N.S.M	N.S.M	N.S.M	-/ -/ -	+/ +/ +	-/ -/ -
**29**	F	N.S.M	**+/-/- (IHC -)**	-/ -/ -	N.S.M	-/ -/ -	+/ +/ +
**30**	F	-/ -/ -	N.S.M	-/ -/ -	N.S.M	-/ -/ -	+/ +/ +
**31**	F	+/ +/ +	-/ -/ -	N.S.M	+/ +/ +	N.S.M	-/ -/ -
**32**	F	-/ -/ -	N.S.M	N.S.M	-/ -/ -	N.S.M	+/ +/ +
**33**	F	-/ -/ -	N.S.M	-/ -/ -	+/ +/ +	-/ -/ -	-/ -/ -
**34**	F	N.S.M	-/ -/ -	+/ +/ +	+/ +/ +	-/ -/ -	N.S.M
**35**	F	N.S.M	-/ -/ -	-/ -/ -	**+/-/-**	**+/-/- (Second H&E -)**	+/ –/ +
**36**	F	N.S.M	-/ -/ -	+/ +/ +	-/ -/ -	N.S.M	-/ -/ -
**37**	F	-/ -/ -	-/ -/ -	N.S.M	-/ -/ -	-/ -/ -	N.S.M

**38**	F	N.S.M	N.S.M	-/ -/ -	-/ -/ -	-/ -/ -	N.S.M
**39**	F	-/ -/ -	N.S.M	-/ -/ -	-/ -/ -	-/ -/ -	+/ +/ +
**40**	F	**+/-/- (Second H&E -)**	-/ -/ -	-/ -/ -	**+/-/- (Second H&E -)**	-/ -/ -	N.S.M
**41**	F	N.S.M	N.S.M	+/ –/ +	–/ –/ –	-/ -/ -	-/ -/ -
**42**	F	-/ -/ -	-/ -/ -	N.S.M	N.S.M	-/ -/ -	-/ -/ - (IHC -)
**43**	F	N.S.M	+/ +/ +	-/ -/ -	-/ -/ -	+/ +/ +	**+/-/-**
**44**	F	N.S.M	N.S.M	+/ +/ +	N.S.M	-/ -/ -	-/ -/ -
**45**	F	-/ -/ -	N.S.M	**+/-/- (IHC -)**	-/ -/ -	-/ -/ -	-/ -/ -
**46**	F	+/ –/ Suspicious (IHC +)	-/ -/ -	N.S.M	-/ -/ -	-/ -/ -	-/ -/ - (Second H&E -)
**47**	F	N.S.M	N.S.M	-/ -/ -	-/ -/ -	+/ +/ +	+/ +/ +
**48**	F	+/ +/ +	-/ -/ -	N.S.M	-/ -/ -	-/ -/ -	+/ –/ +
**49**	F	-/ -/ -	N.S.M	N.S.M	+/ +/ +	N.S.M	-/ -/ -
**50**	F	N.S.M	-/ -/ -	-/ -/ -	N.S.M	**+/-/-**	N.S.M
**51**	F	**+/-/- (Second H&E -)**	N.S.M	N.S.M	N.S.M	-/ -/ -	+/ –/ +
**52**	F	-/ -/ -	–/ –/ –	N.S.M	N.S.M	+/ –/ +	+/ –/ +
**53**	F	-/ -/ -	+/ +/ +	+/ +/ +	**+/-/- (Second H&E -)**	**+/-/- (IHC -)**	N.S.M
**54**	F	N.S.M	N.S.M	-/ -/ -	-/ -/ -	-/ -/ -	N.S.M
**55**	F	N.S.M	-/ -/ -	N.S.M	-/ -/ -	-/ -/ -	N.S.M
**56**	F	N.S.M	N.S.M	N.S.M	+/ +/ +	-/ -/ -	-/ -/ -
**57**	F	+/ +/ +	-/ -/ -	N.S.M	-/ -/ -	-/ -/ -	+/ +/ +
**58**	F	N.S.M	-/ -/ -	+/ +/ +	+/ –/ Suspicious (IHC+)	+/ +/ +	N.S.M
**59**	F	N.S.M	**+/-/- (IHC -)**	-/ -/ -	N.S.M	N.S.M	-/ -/ -
**60**	F	-/ -/ -	N.S.M	+/ +/ +	+/ +/ +	N.S.M	-/ -/ -
**61**	F	-/ -/ -	N.S.M	-/ -/ -	N.S.M	+/ +/ +	-/ -/ -
**62**	F	-/ -/ -	-/ -/ - (IHC -)	-/ -/ -	-/ -/ -	-/ -/ -	-/ -/ -
**63**	F	-/ -/ -	N.S.M	-/ -/ -	-/ -/ -	+/ +/ +	+/ +/ +
**64**	F	N.S.M	-/ -/ -	N.S.M	**+/-/- (Second H&E -)**	-/ -/ -	N.S.M
**65**	F	N.S.M	N.S.M	N.S.M	+/ +/ +	+/ +/ +	-/ -/ -
**66**	F	N.S.M	N.S.M	+/ +/ +	-/ -/ -	N.S.M	+/ +/ +
**67**	F	-/ -/ -	-/ -/ -	+/ –/ +	+/ +/ +	N.S.M	-/ -/ -
**68**	M	-/ -/ -	N.S.M	-/ -/ -	-/ -/ - (IHC -)	**+/-/- (Second H&E -)**	-/ -/ -
**69**	F	+/ +/ +	N.S.M	-/ -/ -	+/ +/ +	-/ -/ -	N.S.M
**70**	F	N.S.M	+/ +/ +	-/ -/ -	+/ –/ Suspicious (IHC+)	-/ -/ -	-/ -/ -
**71**	F	N.S.M	-/ -/ -	+/ +/ +	-/ -/ -	+/ +/ +	-/ -/ -

**72**	F	N.S.M	-/ -/ -	-/ -/ -	+/ +/ +	N.S.M	+/ +/ +
**73**	F	**+/-/- (IHC -)**	+/ +/ +	-/ -/ -	-/ -/ -	+/ –/ +	**+/-/-**
**74**	F	-/ -/ -	-/ -/ - (IHC -)	-/ -/ -	N.S.M	–/ –/ –	-/ -/ -
**75**	F	+/ +/ +	N.S.M	N.S.M	-/ -/ -	+/ +/ +	-/ -/ -
**76**	F	-/ -/ -	N.S.M	N.S.M	-/ -/ -	-/ -/ -	-/ -/ -
**77**	F	N.S.M	-/ -/ -	+/ +/ +	-/ -/ -	+/ +/ +	-/ -/ -
**78**	F	N.S.M	**+/-/- (Second H&E -)**	-/ -/ -	-/ -/ -	-/ -/ -	N.S.M
**79**	F	N.S.M	N.S.M	-/ -/ -	-/ -/ -	+/ +/ +	N.S.M
**80**	F	N.S.M	-/ -/ -	-/ -/ -	N.S.M	+/ –/ +	+/ +/ +
**81**	F	N.S.M	N.S.M	+/ +/ +	N.S.M	-/ -/ -	-/ -/ -
**82**	F	N.S.M	**+/-/- (Second H&E -)**	N.S.M	+/ +/ +	N.S.M	**+/ –/ Suspicious (IHC+)**
**83**	F	N.S.M	-/ -/ - (IHC -)	-/ -/ -	-/ -/ -	N.S.M	-/ -/ -
**84**	F	-/ -/ -	+/ +/ +	-/ -/ -	-/ -/ -	N.S.M	+/ +/ +
**85**	F	N.S.M	N.S.M	-/ -/ - (IHC -)	-/ -/ -	N.S.M	-/ -/ -
**86**	F	N.S.M	N.S.M	-/ -/ -	N.S.M	-/ -/ -	**+/-/- (IHC -)**
**87**	F	**+/-/- (Second H&E -)**	-/ -/ -	N.S.M	-/ -/ -	+/ +/ +	+/ +/ +
**88**	F	-/ -/ -	-/ -/ -	N.S.M	-/ -/ -	N.S.M	-/ -/ -
**89**	F	-/ -/ -	+/ +/ +	+/ +/ +	-/ -/ - (IHC -)	N.S.M	-/ -/ -
**90**	F	N.S.M	-/ -/ -	**+/-/- (Second H&E -)**	N.S.M	-/ -/ -	+/ +/ +
**91**	F	N.S.M	N.S.M	-/ -/ -	+/ +/ +	+/ +/ +	N.S.M
**92**	F	-/ -/ -	-/ -/ - (IHC -)	-/ -/ -	-/ -/ -	-/ -/ -	-/ -/ -
**93**	F	-/ -/ -	N.S.M	-/ -/ -	-/ -/ -	N.S.M	-/ -/ -
**94**	F	**+/+/+**	**-/+/- (Second H&E -)**	**+/+/+**	**-/-/-**	**-/-/- (Second H&E and IHC -)**	**-/-/-**
**95**	F	**+/- /Suspicious (IHC +)**	**-/-/-**	**+/-/+**	**+/-/- (IHC -)**	**-/-/-**	**-/-/-**
**96**	F	**-/-/-**	**-/-/-**	**-/-/+ (Second H&E +) (LVFN)**	**+/+/+**	**+/-/- (IHC -)**	**+/+/+**
**97**	F	**+/+/+**	**-/-/-**	**+/+/+**	**+/+/+**	**-/-/-**	**-/-/-**
**98**	F	**+/+/+**	**+/-/- (Second H&E -)**	**-/-/-**	**-/-/-**	**+/+/+**	**-/-/-**
**99**	F	**-/-/-**	**-/-/-**	**+/-/- (Second H&E -)**	**-/+/+ (LVFN)**	**+/+/+**	**-/-/-**
**100**	F	**+/-/- (Second H&E)**	**+/- /Suspicious (IHC +)**	**+/+/+**	**-/-/-**	**-/-/-**	**+/-/+**

**101**	F	+/+/+	-/-/-	+/+/+	-/-/-	-/-/-	-/-/-
**102**	F	+/+/+	+/-/- (IHC -)	-/-/-	+/+/+	+/+/+	-/-/-
**103**	F	+/-/- (IHC -)	-/-/-	+/-/+	-/-/-	+/+/+	-/-/-
**104**	F	-/-/-	+/+/+	+/-/ Suspicious (Second H&E +)	-/-/-	+/-/- (Second H&E -)	+/+/+
**105**	F	-/-/-	-/+/+ (HVFN)	+/-/- (IHC -)	+/+/+	-/-/-	-/-/+ (LVFN)
**106**	F	-/+/+ (HVFN)	-/-/-	-/+/+ (LVFN)	-/-/-	+/+/+	-/-/-
**107**	F	-/-/-	+/-/- (IHC -)	-/-/-	+/-/+	+/+/+	+/+/+
**108**	F	-/-/-	+/+/+	-/-/-	-/-/-	-/+/+ (HVFN)	-/-/-
**109**	F	-/-/-	+/+/+	+/+/+	-/-/-	-/-/-	-/-/-
**110**	F	+/+/+	-/-/-	-/-/-	-/-/-	-/-/-	+/-/+
**111**	F	-/-/-	+/-/- (IHC -)	-/-/-	+/+/+	-/-/-	+/+/+
**112**	F	+/+/+	-/-/-	+/+/+	+/-/+	-/-/-	-/-/-
**113**	F	-/-/-	+/+/+	-/-/-	+/+/+	-/-/-	-/-/-

**Red**	Positive margins detected by CDP and confirmed by permanent /IHC
**Green**	Negative margins detected by CDP and confirmed by permanent/IHC
**Highlighted blue**	False negatives of CDP
**Highlighted yellow**	False positives of CDP
**N.S.M***	Non-scored margin
**Fro*/Per***	Frozen Pathology/ Permanent Pathology and IHC

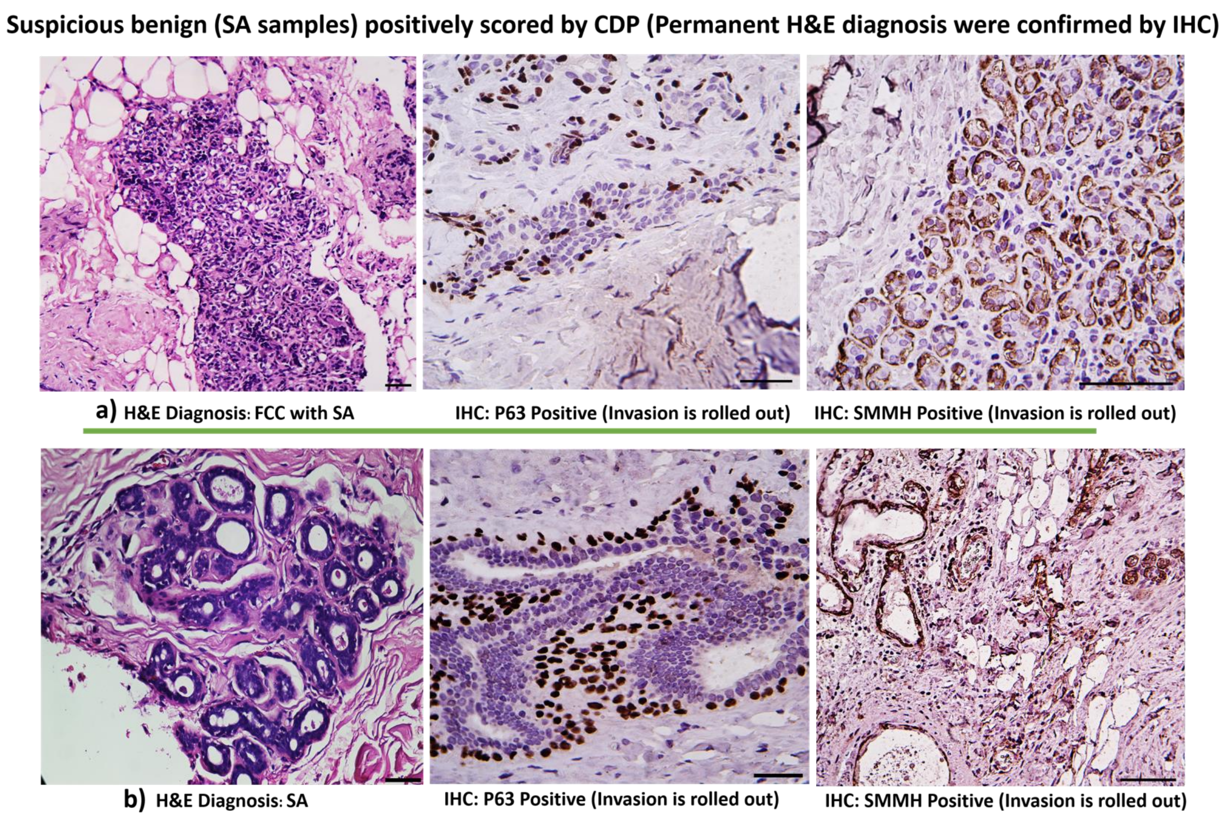

Figure sup4. Permanent H&E of SA lesions were suspicious for the pathologist to be invasive carcinoma. IHC rolled out the invasion due to the expression of SMMH & P63, while CDP scoring was positive. Hence, these margins were FP of CDP a) Patient ID 107 & b) Patient ID 111.

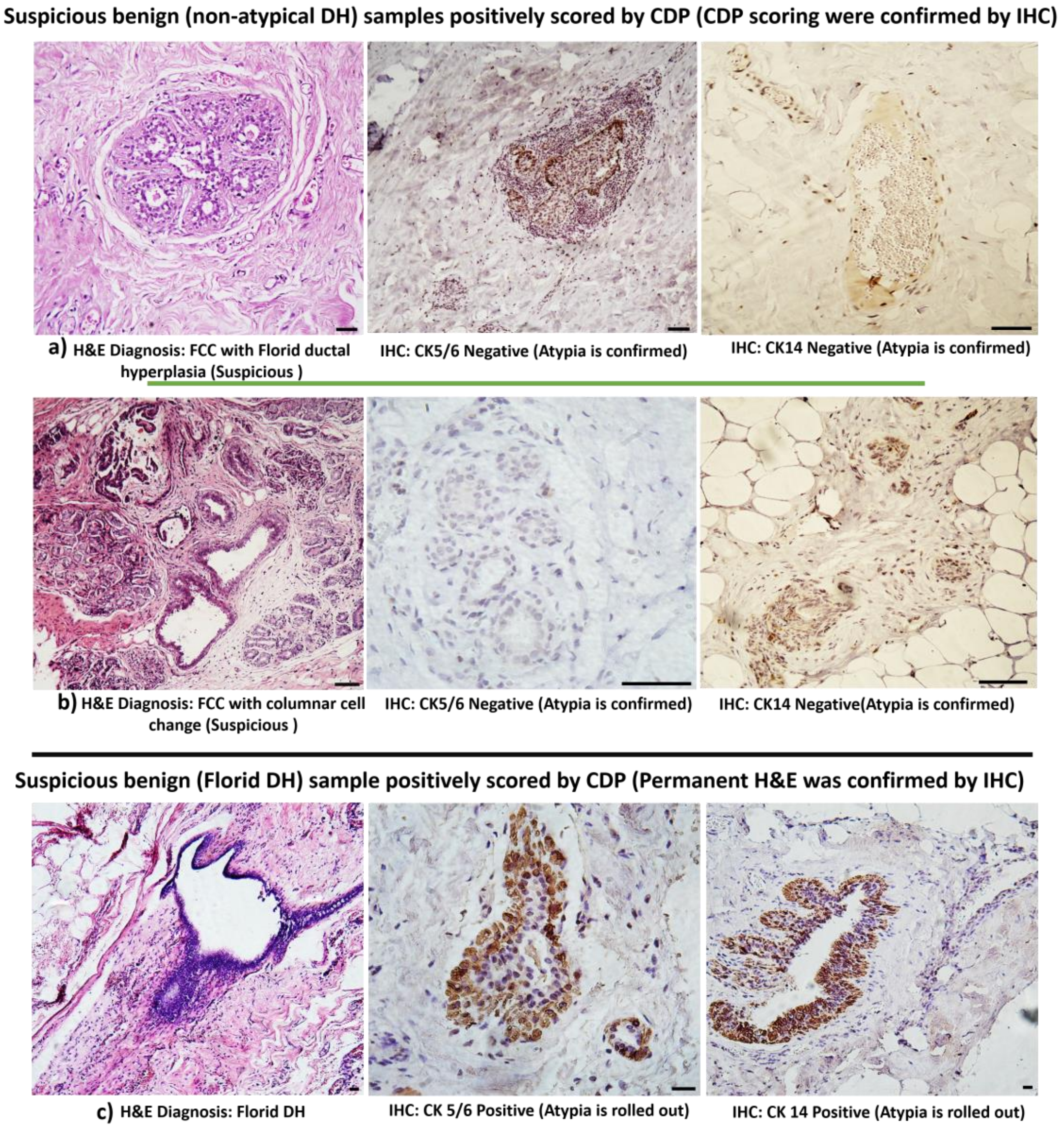

Figure sup5. Permanent H&E of a) FCC with florid DH (Patient ID 14), b) FCC with CCC (Patient ID 96), and c) Florid DH (Patient ID 95) were suspicious and suggestive for atypia through the pathologists' opinions. Thus, CK5/6 and CK14 IHC assays were recommended. Results confirmed the ADH in (a) and (b) due to the non-expression of both CK markers. Hence, the positive scoring of CDP was corroborated. In contrast, atypia was rolled out in (c) due to both CK markers' expression, and CDP scoring was rejected.

Table sup4. Distribution of FPs and FNs per each patient presented in circular diagrams for all 113 patients.

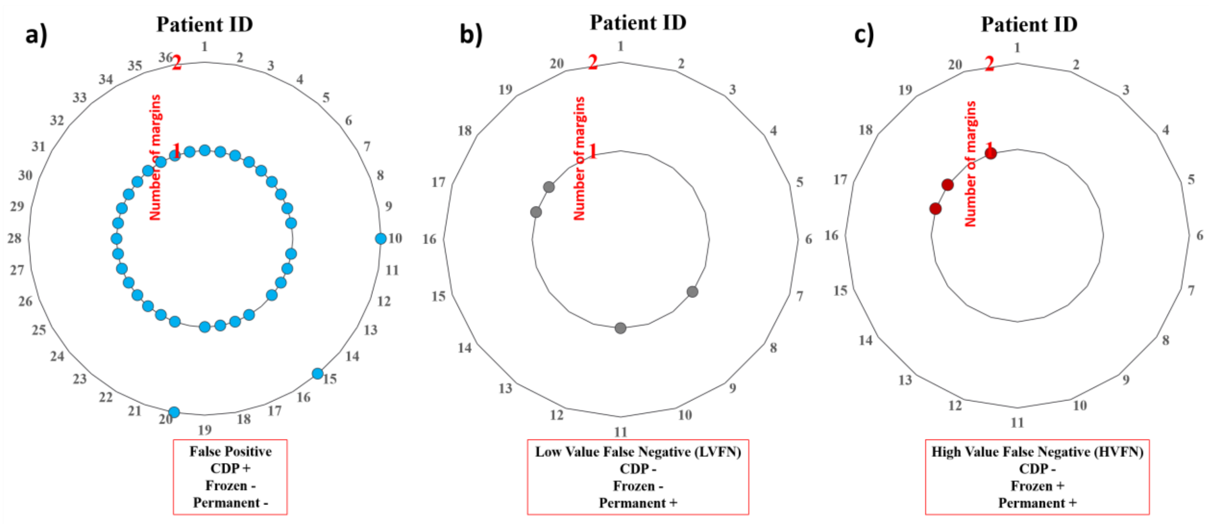

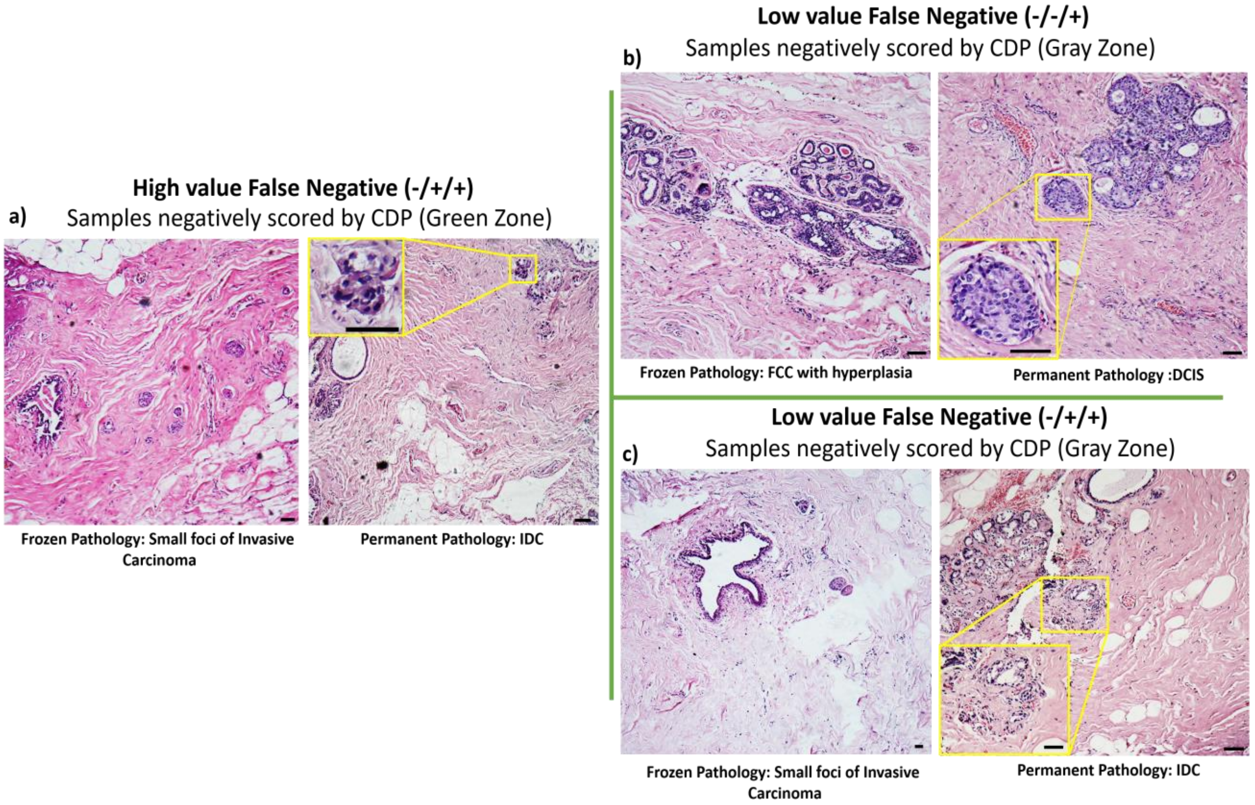

Figure Sup6. Examples from the pathological images of CDP false negatives. a) High-Value False Negative (HVFN) in which Permanent assays diagnosed IDC. The CDP current peak was 80µA. b) Low-Value False Negative (LVFN) in which permanent H&E diagnosed DCIS while the recorded current peak of CDP was 178µA. c) Another (LVFN) of CDP with a current peak of 190µA in which permanent H&E reported the presence of an IDC lesion.

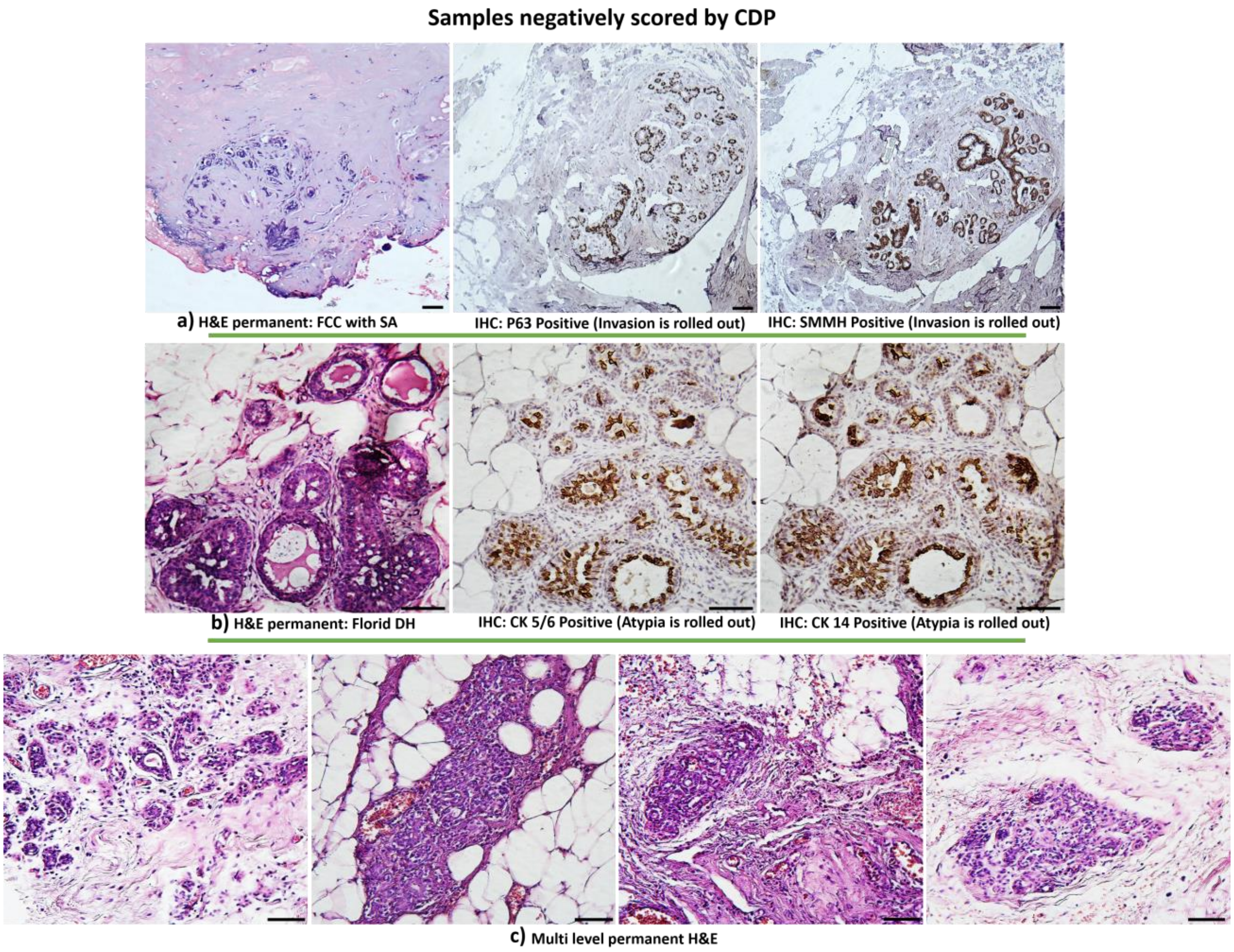

Figure sup7. Re-checking of CFP- margins with IHC and multilevel permanent H&E. Permanent Pathology slides confirmed the negative scores. a) Patient ID 62, b) Patient ID 74, and c) Patient ID 20.

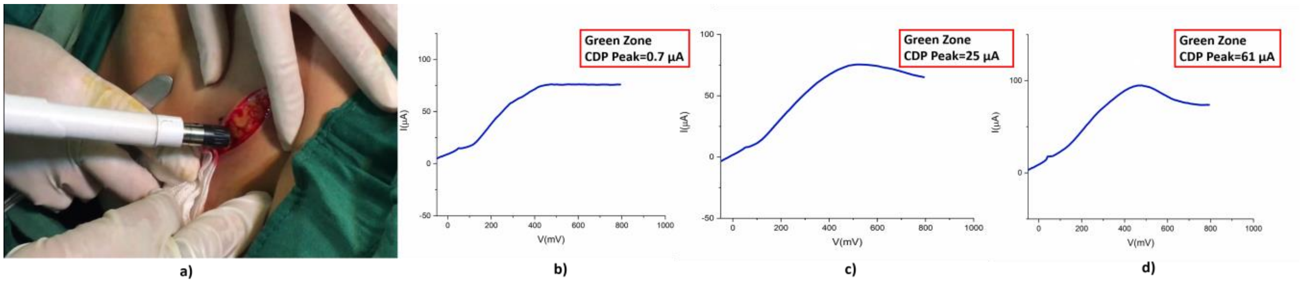

Figure sup8. a) Effect of the wound in CDP responses, (b) immediately, (c) 3 min, and (d) 20 min after wound formation.

S1. Statistical analysis of the clinical reliability of CDP scoring vs. permanent pathology in BCS/mastectomy for 113 patients with different types of breast tumors

Table sup5. CDP scores vs. Permanent Cross tabulation for all EMs and IMs of 113 patients (total number of margins=897) intended in the study

CDP * Permanent Crosstabulation

			Permanent			
			Negative	Positive	10.00	Total
CDP	Negative	Count	500	10	0	510
		% within CDP	98.0%	2.0%	0.0%	100.0%
		% within Permanent	89.3%	3.0%	0.0%	56.9%
	Positive	Count	60	326	1	387
		% within CDP	15.5%	84.2%	0.3%	100.0%
		% within Permanent	10.7%	97.0%	100.0%	43.1%
Total		Count	560	336	1	897
		% within CDP	62.4%	37.5%	0.1%	100.0%
		% within Permanent	100.0%	100.0%	100.0%	100.0%

Sensitivity = 97.00

Specificity = 89.30

Positive Predictive Value = 84.20

Negative Predictive Value = 98.00

Selectivity = 86.60

Table sup6. FROZEN * PERMANENT Cross tabulation for all EMs and IMs of 113 patients (total number of margins=897) intended in the study

Frozen * Permanent Crosstabulation

			Permanent			
			Negative	Positive	10.00	Total
Frozen	Negative	Count	559	79	0	638
		% within Frozen	87.6%	12.4%	0.0%	100.0%
		% within Permanent	99.8%	23.5%	0.0%	71.1%
	Positive	Count	1	257	1	259

	% within Frozen	0.4%	99.2%	0.4%	100.0%
	% within Permanent	0.2%	76.5%	100.0%	28.9%
Total	Count	560	336	1	897
	% within Frozen	62.4%	37.5%	0.1%	100.0%
	% within Permanent	100.0%	100.0%	100.0%	100.0%

Sensitivity = 76.50

Specificity = 99.80

Positive Predictive Value = 99.20

Negative Predictive Value = 87.60

Selectivity = 76.30

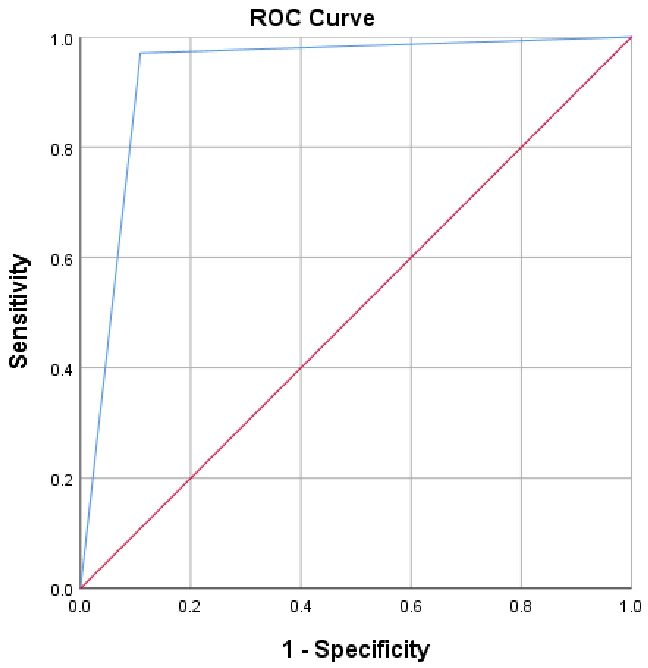

Diagonal segments are produced by ties.

Figure sup9. ROC diagram for CDP vs. permanent results for <u>all EMs and IMs of 113 patients (total number of margins=897) intended in the study</u>

Table sup7. AUC for CDP vs. permanent results for <u>all EMs and IMs of 113 patients (total number of margins=897) intended in the study</u>

Area Under the Curve

Test Result Variable(s): CDP

Area	Std. Error[a]	Asymptotic Sig.[b]	Asymptotic 99% Confidence Interval	
			Lower Bound	Upper Bound
.931	.009	.000	.906	.955

The test result variable(s): CDP has at least one tie between the positive actual state group and the negative actual state group. Statistics may be biased.

a. Under the nonparametric assumption

b. Null hypothesis: true area = 0.5

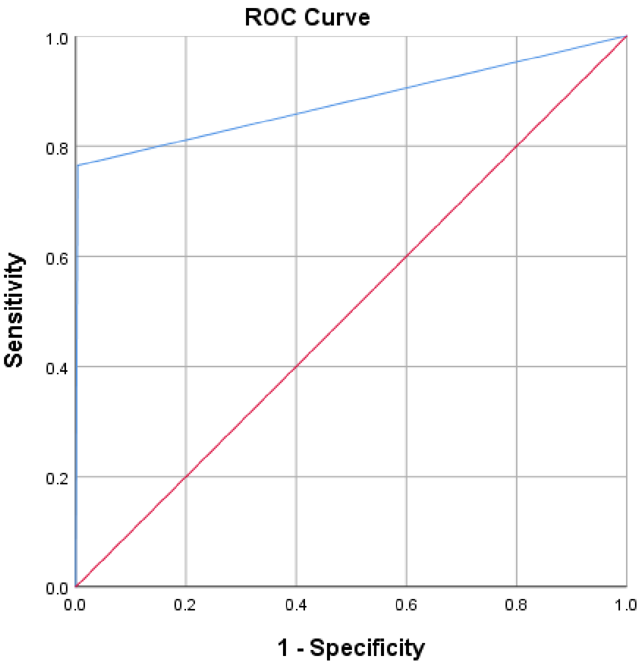

Diagonal segments are produced by ties.

Figure sup10. ROC diagram for frozen vs. permanent results for <u>all EMs and IMs of 113 patients (total number of margins=897) intended in the study</u>

Table sup8. AUC table for frozen vs. permanent results for <u>all EMs and IMs of 113 patients (total number of margins=897) intended in the study</u>

Area Under the Curve

Test Result Variable(s): Frozen

Area	Std. Error[a]	Asymptotic Sig.[b]	Asymptotic 99% Confidence Interval

			Lower Bound	Upper Bound
.881	.014	.000	.844	.917

The test result variable(s): Frozen has at least one tie between the positive actual state group and the negative actual state group. Statistics may be biased.

a. Under the nonparametric assumption

b. Null hypothesis: true area = 0.5

The permanent pathology is considered the gold standard test for the diagnosis of atypical and cancerous specimens. First, the specificity, sensitivity, positive predictive value, negative predictive value, and selectivity of CDP and frozen have been calculated separately. The true and false positive and negative data are shown in detail in Supplementary Tables 5 & 6. It is shown that the sensitivity, selectivity, and accuracy of CDP is better than the frozen.

As a result, to evaluate each of the diagnostic tests, the ROC test has been done to compare each of them with the gold standard test (permanent pathology). As it is shown in ROC and AUC tables for CDP, the area under the curve is 0.931 (P-value<0.0001 and CI99% 0.906-0.955) (Supplementary Figure 9 & Supplementary Table 7), which is higher than 0.9, so the test is appropriate for diagnosis. It has a good balance of sensitivity and specificity.

The same calculation has been done for frozen, and the result shows that (Supplementary Figure 10 & Supplementary Table 8), the area is 0.881 (P-value<0.0001 and CI99% 0.844-0.917), which shows that the test is a reliable diagnostic test.

In conclusion, the CDP has proper sensitivity, selectivity, and accuracy, and it can be used as a diagnostic test of cancerous specimens. Also, the ROC test result shows that the CDP has better results compared to frozen due to the higher area under the curve of CDP (0.931>0.881).

S2. Clinical efficacy of CDP based margin detection/cleaning by the surgeon (3 clinical studies of CDP)

S2.1. Study A; observational study

Table Sup9. CDP scoring on IMs samples vs. pathological diagnoses on reciprocal EMs in an observational study (First study) of 25 additional breast cancer patients. Positive samples indicated with Red (+), negative samples indicated with green (-). During this study, two samples assumed as CDP false (Fourteen false positives and one false negative). NS refers to No-Sampled margins due to the negative score of CDP.

Patient ID	Inferior			Posterior			Anterior			Superior			Medial			Lateral		
	CDP score	Frozen of EM	Permanent of EM	CDP score	Frozen of EM	Permanent of EM	CDP score	Frozen of EM	Permanent of EM	CDP score	Frozen of EM	Permanent of EM	CDP score	Frozen of EM	Permanent of EM	CDP score	Frozen of EM	Permanent of EM
114	-	-	-	-	-	-	+	-	+	-	-	-	-	-	-	+	-	-
115	-	-	-	+	-	-	-	-	-	-	-	-	-	-	-	-	-	-
116	-	-	-	-	-	-	-	-	-	-	-	-	-	-	-	-	-	-
117	+	-	-	-	-	-	-	-	-	+	-	-	-	-	-	-	-	-
118	-	-	-	-	-	-	-	-	-	-	-	-	-	-	-	+	-	-
119	-	-	-	-	-	-	-	-	-	-	-	-	+	-	+	-	-	-
120	-	-	-	-	-	-	-	-	-	-	-	-	-	-	-	-	-	-
121	-	-	-	-	-	-	-	-	-	-	-	-	-	-	-	-	-	-
122	-	-	-	-	-	-	-	-	-	-	-	-	+	-	-	-	-	-
123	+	-	-	-	-	-	+	-	-	-	-	-	-	-	-	-	-	-
124	-	-	-	-	-	-	-	-	-	-	-	-	-	-	-	-	-	-
125	-	-	-	+	-	+	-	-	-	-	-	-	-	-	-	-	-	-
126	-	-	-	-	-	-	-	-	-	-	-	-	-	-	-	-	-	-
127	-	-	-	-	-	-	-	-	-	-	-	-	-	-	-	-	-	-
128	-	-	-	-	-	-	-	-	-	-	-	+	-	-	-	-	-	-
129	-	-	-	-	-	-	-	-	-	-	-	-	-	-	-	-	-	-
130	-	-	-	+	-	-	-	-	-	-	-	-	-	-	-	-	-	-
131	-	-	-	-	-	-	-	-	-	-	-	-	+	-	-	-	-	-
132	-	-	-	-	-	-	-	-	-	-	-	-	-	-	-	-	-	-
133	+	-	-	-	-	-	-	-	-	-	-	-	-	-	-	-	-	-
134	-	-	-	-	-	-	-	-	-	-	-	-	-	-	-	-	-	-
135	-	-	-	-	-	-	-	-	-	-	-	-	-	-	-	-	-	-
136	-	-	-	-	-	-	-	-	-	-	-	-	+	-	-	+	-	-
137	-	-	-	-	-	-	-	-	-	-	-	-	-	-	-	-	-	-

138	+	-	+	-	-	-	+	-	-	-	-	-	-	-	-	-	-	-

Table sup10. CDP scores vs. Permanent Cross tabulation for total 150 EM and IM margins on 25 patients in the first study.

CDP * EMPermanent Crosstabulation

			EMPermanent		
			Negative	Positive	Total
CDP	Negative	Count	131	1	132
		% within CDP	99.2%	0.8%	100.0%
		% within EMPermanent	90.3%	20.0%	88.0%
	Positive	Count	14	4	18
		% within CDP	77.8%	22.2%	100.0%
		% within EMPermanent	9.7%	80.0%	12.0%
Total		Count	145	5	150
		% within CDP	96.7%	3.3%	100.0%
		% within EMPermanent	100.0%	100.0%	100.0%

Sensitivity = 80.00

Specificity = 90.3

Positive Predictive Value = 22.20

Negative Predictive Value = 99.20

Selectivity = 72.22

Table sup11. FROZEN * PERMANENT Cross tabulation for total 150 EM and IM margins on 25 patients in the first study.

Frozen * Permanent of EM Crosstabulation

			Permanent of EM		
			Negative	Positive	Total
Frozen	Negative	Count	145	5	150
		% within Frozen	96.7%	3.3%	100.0%
		% within Permanent of EM	100.0%	100.0%	100.0%

Total	Count	145	5	150
	% within Frozen	96.7%	3.3%	100.0%
	% within Permanent of EM	100.0%	100.0%	100.0%

Sensitivity = 0.00

Specificity = 100.00%

Positive Predictive Value = 0.00

Negative Predictive Value = 96.70%

Selectivity = 0.00

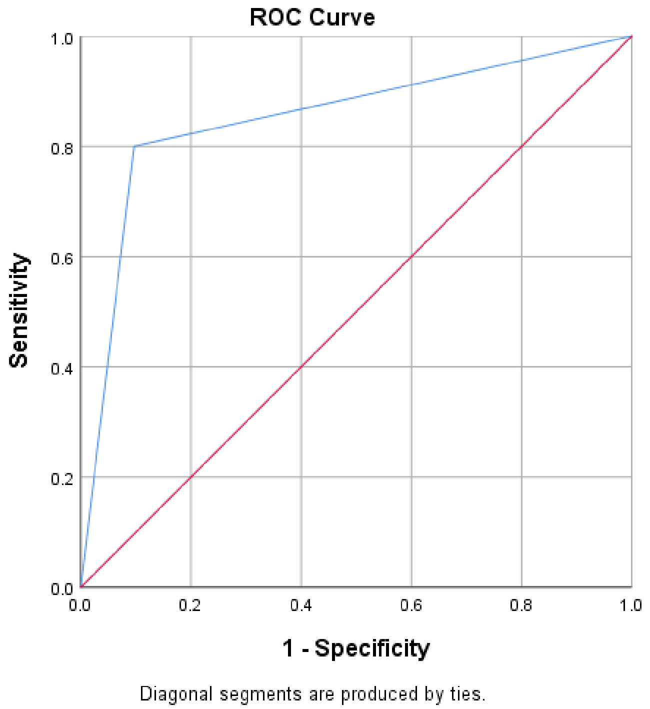

Figure sup11. Receiver Operating Characteristic (ROC) diagram for CDP vs. permanent of EM results for total 150 EM and IM margins on 25 patients in the first study.

Table sup12. Area Under the Curve (AUC) for CDP vs. permanent of EM for total 150 EM and IM margins on 25 patients in the first study.

Area Under the Curve

Test Result Variable(s): CDP

Area	Std. Error[a]	Asymptotic Sig.[b]	Asymptotic 99% Confidence Interval	
			Lower Bound	Upper Bound
.852	.105	.008	.580	1.000

The test result variable(s): CDP has at least one tie between the positive actual state group and the negative actual state group. Statistics may be biased.

a. Under the nonparametric assumption

b. Null hypothesis: true area = 0.5

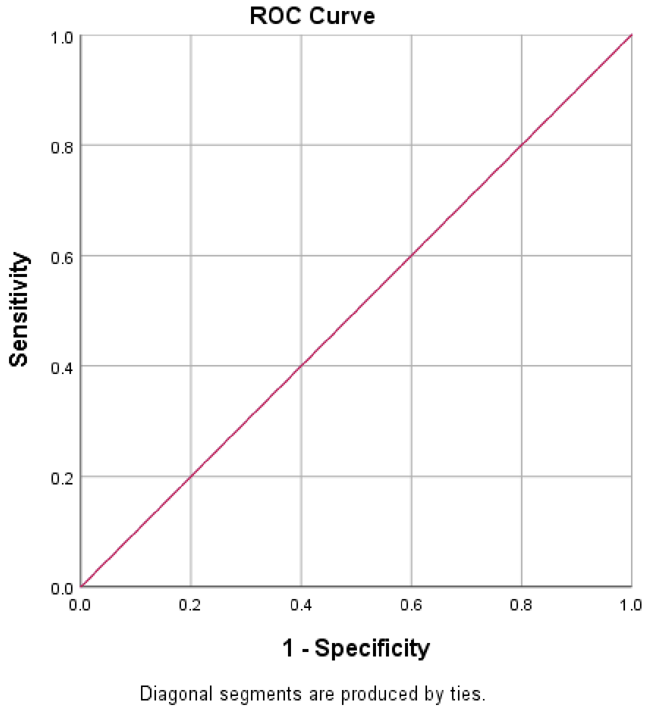

Figure sup12. Receiver operating characteristic (ROC) diagram for Frozen vs. permanent of EM for total 150 EM and IM margins on 25 patients in the first study.

Table sup13. Area under the Curve (AUC) table for Frozen vs. permanent of EM for total 150 EM and IM margins on 25 patients in the first study.

Area Under the Curve

Test Result Variable(s): Frozen

Area	Std. Error[a]	Asymptotic Sig.[b]	Asymptotic 99% Confidence Interval	
			Lower Bound	Upper Bound
.500	.132	1.000	.161	.839

The test result variable(s): Frozen has at least one tie between the positive actual state group and the negative actual state group. Statistics may be biased.

a. Under the nonparametric assumption

b. Null hypothesis: true area = 0.5

The permanent pathology is considered the gold standard test for the diagnosis of atypical and cancerous specimens. First, the specificity, sensitivity, positive predictive value, negative predictive value, and selectivity of CDP and frozen have been calculated separately. The true and false positive and negative data are shown in detail in Supplementary Tables 10 & 11. It is shown that the sensitivity, accuracy, and selectivity of CDP is better than the frozen.

As a result, to evaluate each of the diagnostic tests, the ROC test has been done to compare each of them with the gold standard test (permanent pathology). As it is shown in ROC and AUC table for CDP, the area under the curve is 0.852 (P-value<0.008 and CI99% 0.580-1.000) (Supplementary Figure 11 & Supplementary Table 12), which is higher than 0.9. Hence, the test is appropriate for diagnosis, and it has a good balance of sensitivity and specificity.

The same calculation has been done for frozen, and the result shows that (Supplementary Figure 12 & Supplementary Table 13), the area is 0.5 (P-value>0.01 and CI99% 0.161-0.839), which shows that the test is not a reliable diagnostic test. It has not a good balance of sensitivity and specificity.

In conclusion, the CDP has proper sensitivity, accuracy, and selectivity, and it can be used as a diagnostic test of cancerous specimens. Also, the ROC test result shows that the CDP has better results compared to frozen due to the higher area under the curve of CDP (0.852>0.500).

S2.2. Study B; independent role of CDP in an interventional study

Table Sup14. CDP scoring on IMs samples vs. pathological diagnoses on reciprocal EMs in the second study of 25 additional breast cancer patients. Positive samples indicated with Red (+), negative samples indicated with green (-). During this study, two samples assumed as CDP false (twelve false positives and one false negative). NS refers to No-Sampled margins due to the negative score of CDP.

Patient ID	Inferior				Posterior				Anterior				Superior				Medial				Lateral			
	Frozen of EM	CDP prediction on IM	Permanent of CDP	Permanent of EM	Frozen of EM	CDP prediction on IM	Permanent of CDP	Permanent of EM	Frozen of EM	CDP prediction on IM	Permanent of CDP	Permanent of EM	Frozen of EM	CDP prediction on IM	Permanent of CDP	Permanent of EM	Frozen of EM	CDP prediction on IM	Permanent of CDP	Permanent of EM	Frozen of EM	CDP prediction on IM	Permanent of CDP	Permanent of EM
139	-	-	NS	-	-	+	-	-	-	-	NS	-	-	-	NS	-	-	-	NS	-	-	-	NS	-
140	+	+	+	+	-	-	NS	-	-	-	NS	-	+	+	+	+	+	+	+	+	-	-	NS	-
141	-	-	NS	-	-	-	NS	-	-	-	NS	-	-	-	NS	-	-	-	NS	-	-	-	NS	-
142	-	-	NS	-	-	-	NS	-	-	-	NS	-	-	-	NS	-	-	-	NS	-	-	+	-	-
143	-	-	NS	-	-	-	NS	-	-	-	NS	-	-	+	+	-	-	-	NS	-	-	+	+	-
144	-	+	-	-	-	-	NS	-	-	+	-	-	-	-	NS	-	-	-	NS	-	-	-	NS	-
145	-	-	NS	-	-	-	NS	-	-	+	+	+	-	-	NS	-	-	+	+	-	-	-	NS	-
146	-	-	NS	-	-	-	NS	-	-	-	NS	-	-	-	NS	-	-	-	NS	-	-	-	NS	-
147	-	-	NS	-	-	-	NS	-	-	-	NS	-	-	-	NS	-	-	+	+	-	-	-	NS	-
148	-	-	NS	-	-	-	NS	-	-	-	NS	-	-	-	NS	-	-	-	NS	-	-	-	NS	-
149	-	+	+	-	-	-	NS	-	-	-	NS	-	-	+	-	-	-	-	NS	-	-	-	NS	-
150	-	-	NS	-	-	-	NS	-	-	-	NS	-	-	-	NS	-	-	+	-	-	-	-	NS	-
151	-	-	NS	-	-	-	NS	-	-	-	NS	-	-	-	NS	-	-	-	NS	-	-	+	+	-
152	-	-	NS	-	-	-	NS	-	-	-	NS	-	-	-	NS	-	-	-	NS	-	-	-	NS	-
153	-	-	NS	-	-	-	NS	-	-	-	NS	-	-	-	NS	-	-	-	NS	-	-	-	NS	-
154	-	+	+	-	-	+	-	-	-	-	NS	-	-	-	NS	-	-	-	NS	-	-	-	NS	-
155	-	-	NS	-	-	-	NS	-	-	+	-	-	-	-	NS	-	-	-	NS	-	-	+	-	-
156	-	-	NS	-	-	-	NS	-	-	-	NS	-	-	-	NS	-	-	-	NS	-	-	-	NS	-
157	-	-	NS	-	-	-	NS	-	-	-	NS	-	-	-	NS	-	-	-	NS	-	-	-	NS	-
158	-	-	NS	-	-	-	NS	-	-	-	NS	-	+	-	NS	+	-	-	NS	-	-	-	NS	-
159	-	-	NS	-	-	-	NS	-	-	-	NS	-	-	-	NS	-	-	-	NS	-	-	-	NS	-
160	+	+	+	+	-	-	NS	-	-	+	-	-	-	-	NS	-	-	-	NS	-	-	-	NS	-
161	-	+	+	-	-	-	NS	-	-	-	NS	-	-	-	NS	-	-	-	NS	-	-	+	+	-
162	-	-	NS	-	-	-	NS	-	-	-	NS	-	-	-	NS	-	-	-	NS	-	-	+	-	-

163	-	+	-	-	-	-	NS	-	-	-	NS	-	-	-	NS	-	-	+	+	+	-	-	NS	-

Table sup15. CDP scores vs. Permanent Cross tabulation for total 150 EM and IM margins on 25 patients in the second study.

CDP * CDPPermanent Crosstabulation

			CDPPermanent		Total
			Negative	Positive	
CDP	Negative	Count	122	1	123
		% within CDP	99.2%	0.8%	100.0%
		% within CDPPermanent	91.0%	6.3%	82.0%
	Positive	Count	12	15	27
		% within CDP	44.4%	55.6%	100.0%
		% within CDPPermanent	9.0%	93.8%	18.0%
Total		Count	134	16	150
		% within CDP	89.3%	10.7%	100.0%
		% within CDPPermanent	100.0%	100.0%	100.0%

Sensitivity = 93.80

Specificity = 91.00

Positive Predictive Value = 55.60

Negative Predictive Value = 99.20

Selectivity = 86.00

Table sup16. FROZEN * PERMANENT Cross tabulation for total 150 EM and IM margins on 25 patients in the second study.

Frozen * Permanent of EM Crosstabulation

			Permanent of EM		Total
			Negative	Positive	

Frozen	Negative	Count	134	11	145
		% within Frozen	92.4%	7.6%	100.0%
		% within Permanent of EM	100.0%	68.8%	96.7%
	Positive	Count	0	5	5
		% within Frozen	0.0%	100.0%	100.0%
		% within Permanent of EM	0.0%	31.3%	3.3%
Total		Count	134	16	150
		% within Frozen	89.3%	10.7%	100.0%
		% within Permanent of EM	100.0%	100.0%	100.0%

Sensitivity = 31.30

Specificity = 100.00

Positive Predictive Value = 100.00

Negative Predictive Value = 92.40

Selectivity = 31.30

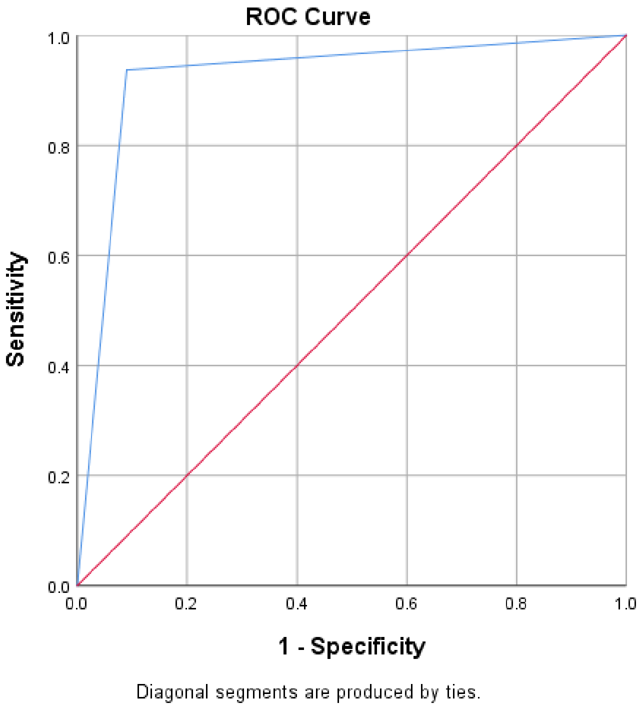

Figure sup13. ROC diagram for CDP vs. permanent of EM results for total 150 EM and IM margins on 25 patients in the second study.

Table sup17. AUC for CDP vs. permanent of EM for total 150 EM and IM margins on 25 patients in the second study.

Area Under the Curve

Test Result Variable(s): CDP

Area	Std. Error[a]	Asymptotic Sig.[b]	Asymptotic 99% Confidence Interval	
			Lower Bound	Upper Bound
.924	.038	.000	.826	1.000

The test result variable(s): CDP has at least one tie between the positive actual state group and the negative actual state group. Statistics may be biased.

a. Under the nonparametric assumption

b. Null hypothesis: true area = 0.5

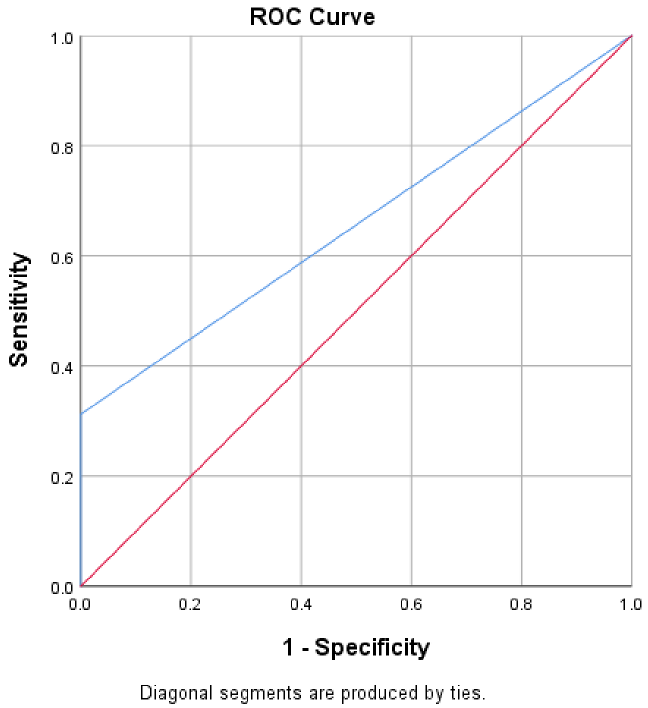

Figure sup14. ROC diagram for Frozen vs. permanent of EM for total 150 EM and IM margins on 25 patients in the second study.

Table sup18. AUC table for Frozen vs. permanent of EM for total 150 EM and IM margins on 25 patients in the second study.

Area Under the Curve

Test Result Variable(s): Frozen EM

Area	Std. Error[a]	Asymptotic Sig.[b]	Asymptotic 99% Confidence Interval	
			Lower Bound	Upper Bound
.656	.085	.041	.437	.876

The test result variable(s): Frozen EM has at least one tie between the positive actual state group and the negative actual state group. Statistics may be biased.

a. Under the nonparametric assumption

b. Null hypothesis: true area = 0.5

The permanent pathology is considered the gold standard test for the diagnosis of atypical and cancerous specimens. First, the specificity, sensitivity, positive predictive value, negative predictive value, and selectivity of CDP and frozen have been calculated separately. The true and

false positive and negative data are shown in detail in Supplementary Tables 15&16. It is shown that the sensitivity, accuracy, and selectivity of CDP is better than the frozen.

As a result, to evaluate each of the diagnostic tests, the ROC test has been done to compare each of them with the gold standard test (permanent pathology). As it is shown in ROC and AUC table for CDP, the area under the curve is 0.924 (P-value<0.00001 and CI99% 0.826-1.000) (Supplementary Figure 13 & Supplementary Table 17), which is higher than 0.9. Hence, the test is appropriate for diagnosis, and it has not a good balance of sensitivity and specificity.

The same calculation has been done for frozen, and the result shows that (Supplementary Figure 14 & Supplementary Table 18), the area is 0.656 (P-value>0.01 and CI99% 0.437-0.876), which shows that the test is not a reliable diagnostic test. It has not a good balance of sensitivity and specificity.

In conclusion, the CDP has proper sensitivity, accuracy, and selectivity, and it can be used as a diagnostic test of cancerous specimens. Also, the ROC test result shows that the CDP has better results compared to frozen due to the higher area under the curve of CDP (0.924>0.656).

S2.3. Study C; Complementary role of CDP in an interventional study

Table Sup19. CDP scoring on IMs samples vs. pathological diagnoses on reciprocal EMs in the third study of 25 additional breast cancer patients. Positive samples indicated with Red (+), negative samples indicated with green (-). During this study, one sample assumed as CDP false (eleven false positives). NS refers to No-Sampled margins due to the negative score of CDP. Two samples had been positively scored by CDP, weren't confirmed in Frozen re-evaluation of the EM reciprocal, but confirmed in Permanent pathology of EM patient's ID 183 and 187. NS refers to No-Sampled margins.

P	Inferior	Posterior	Anterior	Superior	Medial	Lateral

	CDP score	Frozen re-evaluation of EM	Permanent of EM	CDP score	Frozen re-evaluation of EM	Permanent of EM	CDP score	Frozen re-evaluation of EM	Permanent of EM	CDP score	Frozen re-evaluation of EM	Permanent of EM	CDP score	Frozen re-evaluation of EM	Permanent of EM	CDP score	Frozen re-evaluation of EM	Permanent of EM
164	+	+	+	-	NS	-	+	NS	-	-	NS	-	-	NS	-	-	NS	-
165	-	NS	-	-	NS	-	-	NS	-	-	NS	-	-	NS	-	-	NS	-
166	-	NS	-	-	NS	-	-	NS	-	+	+	+	-	NS	-	-	NS	-
167	-	NS	-	-	NS	-	-	NS	-	-	NS	-	-	NS	-	-	NS	-
168	-	NS	-	-	NS	-	-	NS	-	-	NS	-	-	NS	-	-	NS	-
169	-	NS	-	-	NS	-	-	NS	-	-	NS	-	+	NS	-	+	NS	-
170	-	NS	-	+	+	+	-	NS	-	-	NS	-	-	NS	-	-	NS	-
171	+	NS	-	-	NS	-	-	NS	-	-	NS	-	-	NS	-	-	NS	-
172	-	NS	-	-	NS	-	-	NS	-	-	NS	-	-	NS	-	-	NS	-
173	-	NS	-	+	NS	-	+	+	+	-	NS	-	-	NS	-	-	NS	-
174	-	NS	-	-	NS	-	-	NS	-	-	NS	-	-	NS	-	-	NS	-
175	-	NS	-	-	NS	-	-	NS	-	-	NS	-	-	NS	-	-	NS	-
176	-	NS	-	-	NS	-	-	NS	-	+	NS	-	-	NS	-	-	NS	-
177	-	NS	-	-	NS	-	-	NS	-	-	NS	-	-	NS	-	-	NS	-
178	+	NS	-	-	NS	-	-	NS	-	-	NS	-	-	NS	-	-	NS	-
179	-	NS	-	-	NS	-	-	NS	-	+	+	+	-	NS	-	-	NS	-
180	-	NS	-	-	NS	-	-	NS	-	-	NS	-	+	NS	-	-	NS	-
181	-	NS	-	-	NS	-	-	NS	-	-	NS	-	-	NS	-	-	NS	-
182	-	NS	-	-	NS	-	+	NS	-	-	NS	-	-	NS	-	-	NS	-
183	-	NS	-	-	NS	-	-	NS	-	-	NS	-	+	-	+	-	NS	-
184	-	NS	-	-	NS	-	-	NS	-	-	NS	-	-	NS	-	-	NS	-
185	-	NS	-	+	+	+	-	NS	-	-	NS	-	-	NS	-	-	NS	-
186	-	NS	-	-	NS	-	-	NS	-	+	NS	-	-	NS	-	-	NS	-
187	-	NS	-	-	NS	-	-	NS	-	-	NS	-	-	NS	-	+	-	+
188	-	NS	-	+	NS	-	-	NS	-	-	NS	-	-	NS	-	-	NS	-

Table sup20. CDP scores vs. Permanent Cross tabulation for total 150 EM and IM margins on 25 patients in the third study

CDP * EMPermanent Crosstabulation

			EMPermanent		
			Negative	Positive	Total
CDP	Negative	Count	131	0	131
		% within CDP	100.0%	0.0%	100.0%

		% within EMPermanent	92.3%	0.0%	87.3%
	Positive	Count	11	8	19
		% within CDP	57.9%	42.1%	100.0%
		% within EMPermanent	7.7%	100.0%	12.7%
Total		Count	142	8	150
		% within CDP	94.7%	5.3%	100.0%
		% within EMPermanent	100.0%	100.0%	100.0%

Sensitivity = 100.00

Specificity = 92.30

Positive Predictive Value = 42.1.90

Negative Predictive Value = 100.00

Selectivity = 92.30

Table sup21. FROZEN * PERMANENT Cross tabulation for total 150 EM and IM margins on 25 patients in the third study.

Frozen conventional evaluation * Permanent EM Crosstabulation

			Permanent of EM		
			Negative	Positive	Total
Frozen conventional evaluation	Negative	Count	142	8	150
		% within Frozen conventional evaluation	94.7%	5.3%	100.0%
		% within Permanent of EM	100.0%	100.0%	100.0%
Total		Count	142	8	150
		% within Frozen conventional evaluation	94.7%	5.3%	100.0%
		% within Permanent EM	100.0%	100.0%	100.0%

Sensitivity = 0.00

Specificity = 100.00

Positive Predictive Value = 0.00

Negative Predictive Value = 94.70

Selectivity = 0.00

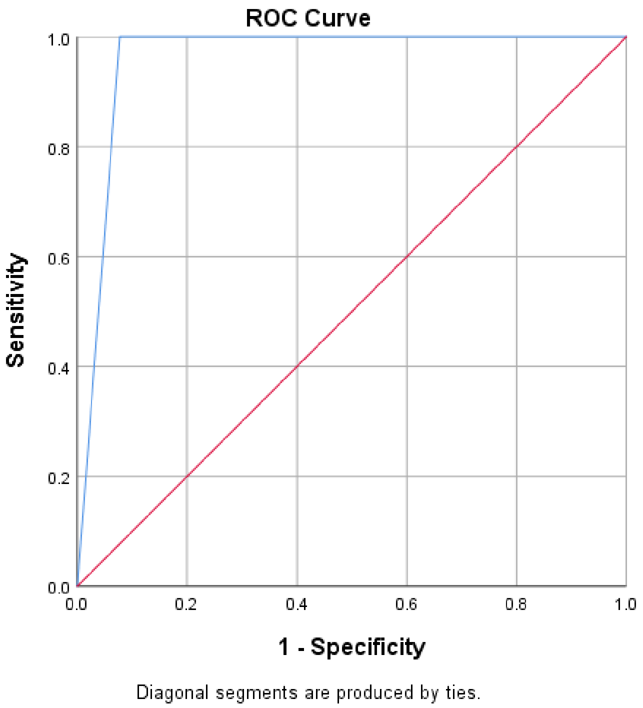

Figure sup15. ROC diagram for CDP vs. permanent of EM results for total 150 EM and IM margins on 25 patients in the third study.

Table sup22. AUC for CDP vs. permanent of EM for total 150 EM and IM margins on 25 patients in the third study.

Area Under the Curve

Test Result Variable(s): CDP

Area	Std. Error[a]	Asymptotic Sig.[b]	Asymptotic 99% Confidence Interval	
			Lower Bound	Upper Bound
.961	.016	.000	.921	1.000

The test result variable(s): CDP has at least one tie between the positive actual state group and the negative actual state group. Statistics may be biased.

a. Under the nonparametric assumption

b. Null hypothesis: true area = 0.5

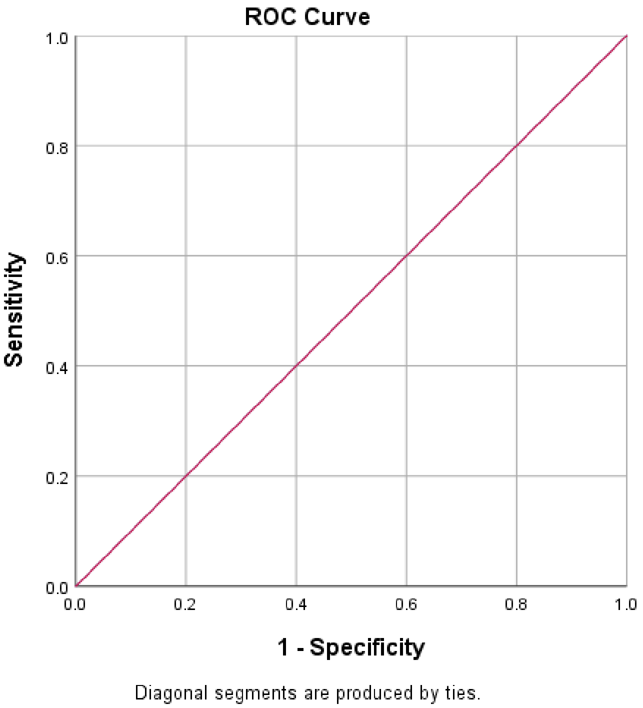

Figure sup16. ROC diagram for Frozen vs. permanent of EM for total 150 EM and IM margins on 25 patients in the third study.

Table sup23. AUC table for Frozen vs. permanent of EM for total 150 EM and IM margins on 25 patients in the third study.

Area Under the Curve

Test Result Variable(s): Frozen conventional evaluation

			Asymptotic 99% Confidence Interval	
Area	Std. Error[a]	Asymptotic Sig.[b]	Lower Bound	Upper Bound
.500	.105	1.000	.229	.771

The test result variable(s): Frozen conventional evalution has at least one tie between the positive actual state group and the negative actual state group. Statistics may be biased.

a. Under the nonparametric assumption

b. Null hypothesis: true area = 0.5

The permanent pathology is considered the gold standard test for the diagnosis of atypical and cancerous specimens. First, the specificity, sensitivity, positive predictive value, negative

predictive value, and selectivity of CDP and frozen have been calculated separately. The true and false positive and negative data are shown in detail in Supplementary Tables 22 & 21. It is shown that the sensitivity, accuracy, precision, and selectivity of CDP is better than the frozen.

As a result, to evaluate each of the diagnostic tests, the ROC test has been done to compare each of them with the gold standard test (permanent pathology). As it is shown in ROC and AUC table for CDP, the area under the curve is 0.961 (P-value<0.00001 and CI99% 0.921-1.000) (Supplementary Figure 15 & Supplementary Table 22), which is higher than 0.9. Hence, the test is appropriate for diagnosis. It has a good balance of sensitivity and specificity.

The same calculation has been done for frozen, and the result shows that (Supplementary Figure 16 & Supplementary Table 23), the area is 0.500 (P-value>0.01 and CI99% 0.229-0.771), which shows that the test is not a reliable diagnostic test. It has not a good balance of sensitivity and specificity.

In conclusion, the CDP has proper sensitivity, accuracy, precision, and selectivity, and it can be used as a diagnostic test of cancerous specimens. Also, the ROC test result shows that the CDP has better results compared to frozen due to the higher area under the curve of CDP (0.961>0.500). The accurate amount of each index is shown in the tables.

S3. Biological evidence associated with false positives of CDP

The number of falsely scored samples by CDP is much lower than truly scored ones (FP= (60/897) = 6.7% and FN= (10/897) = 1.11% for total IMs and EMs of 113 patients (Total IMs =491, total EMs=406). Glycolytic pathways might be activated in falsely scored samples, while those lesions' pathological transformation to atypical or neoplastic states still were not observable. Further

analyses by HIF1 alpha IHC marker showed that the samples which have been positively scored by CDP (ADH) but were not confirmed by permanent (florid/moderate DH) (24/60=40%) (Supplementary Figure 17) expressed considerable levels of HIF1 alpha (12/24=50%). However, by applying HIF1 alpha assays on UDH samples that had been truly scored negative by CDP (due to the confirmation of permanent), we observed negligible expressed levels of HIF1 alpha on those lesions (Supplementary Figure 18). Hence some of false positives of CDP with overexpression of HIF1 alpha might be suspicious to be pre-cancerous. (4/60) = 6.7% of the false-positive samples were SA lesions. SA lesions are high impact lesions for pathologists and sometimes recommended for dissection [5]. The main challenge in SA lesions is that they might be missed with invasive carcinoma and only the IHC (SMMH & P63) are the distinguishing factors for such diagnosis. In this case, permanent/IHC assay can declare final diagnose on such lesions (they cannot be decisively diagnosed by frozen assay). This would further emphasize the impact of CDP scoring in false regions. Low percent (2/60=3.3%) of the false positive (miss classified) samples were normal breasts, which we required further evaluations on them.

Also, due to the small dissected sample size of CDP ($3\times3\times4$ mm^3) because of its positive score, which was not detected as a preneoplastic or neoplastic lesion by a pathologist, removal of these samples is not harmful to the patient or disrupt the diagnosis process. Even due to the presence of high-risk pre-cancer lesions such as columnar cell hyperplasia and columnar cell change along with apocrine metaplasia lesions in the margins that formed most of our false positives (30/60=50%), it is useful for the patient to remove them [6],[7],[8].

In the case of misclassified samples in false negatives, high value and low-value false negatives must be discussed separately. HVFNs are very rare (just 3/491 of the samples for IMs), which are

the system's main limitation. Small foci of the target lesion or distance of the sensing needle from the targeted lesion might induce such false. Also, the necrotic/apoptotic states of those lesions, which affect their metabolism, might suppress their hypoxic functions. This concept is our program in the future to be analyzed on any probable HVFN samples by P53 IHC assay. As LVFNs were supported by CDP to be dissected, these lesions are not crucial false scores.

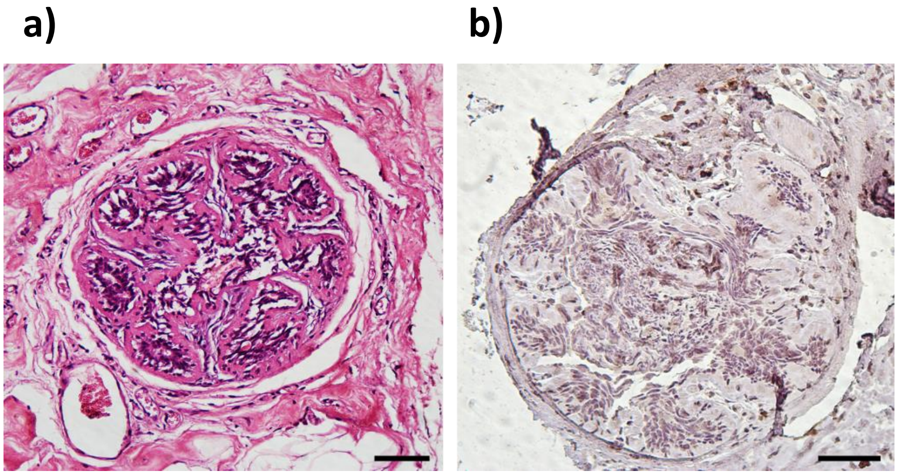

Figure sup17. Florid DH lesion falsely scored positive by CDP while HIF was expressed considerable levels of HIF1 alpha, a) Permanent H&E, and b) HIF1α

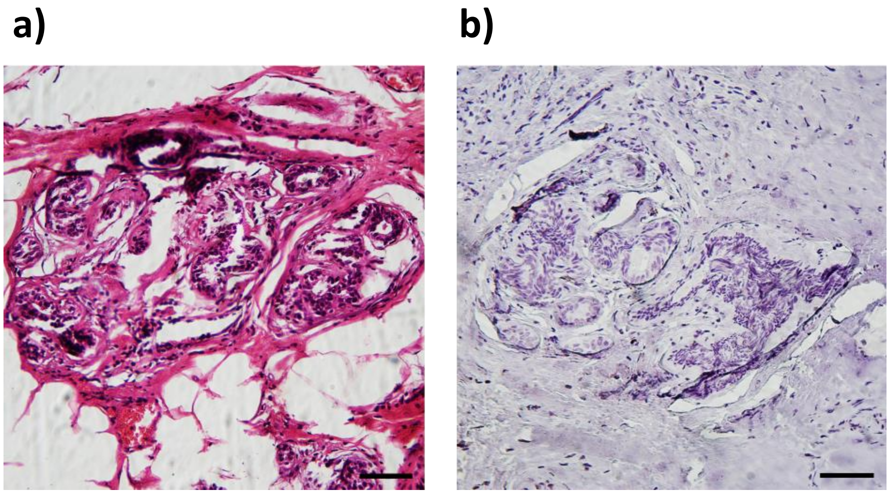

Figure sup18. Florid DH lesion scored negative by CDP and HIF was not expressed, a) Permanent H&E and, b) HIF1α

Moreover, during our investigations, we found phenomena named “Field Effect” in which an entire area of tissue appears normal but has been “cancerized” by hydrogen peroxide and oxidative stress [9] (Supplementary Figure 19) secreted by tumor-associated fibroblasts and adjacent tumor microenvironment, which might be in a great correlation with some of our false positives. We might have detected the H_2O_2 in the environment of normal stromal margins had been cancerized through “field effect” but revealed no pathological signs of cancerization. As most of CDP’s FPs are low-risk benign lesions, such as SA or FCC with CCC, this probability might be valuable to investigate. Approving this phenomenon requires studying the expression of “Caveolin-1” associated transcriptomes [10], which are under our investigations for the future. Therefore, we still consider such miss-classified positive results as false positive responses of CDP.

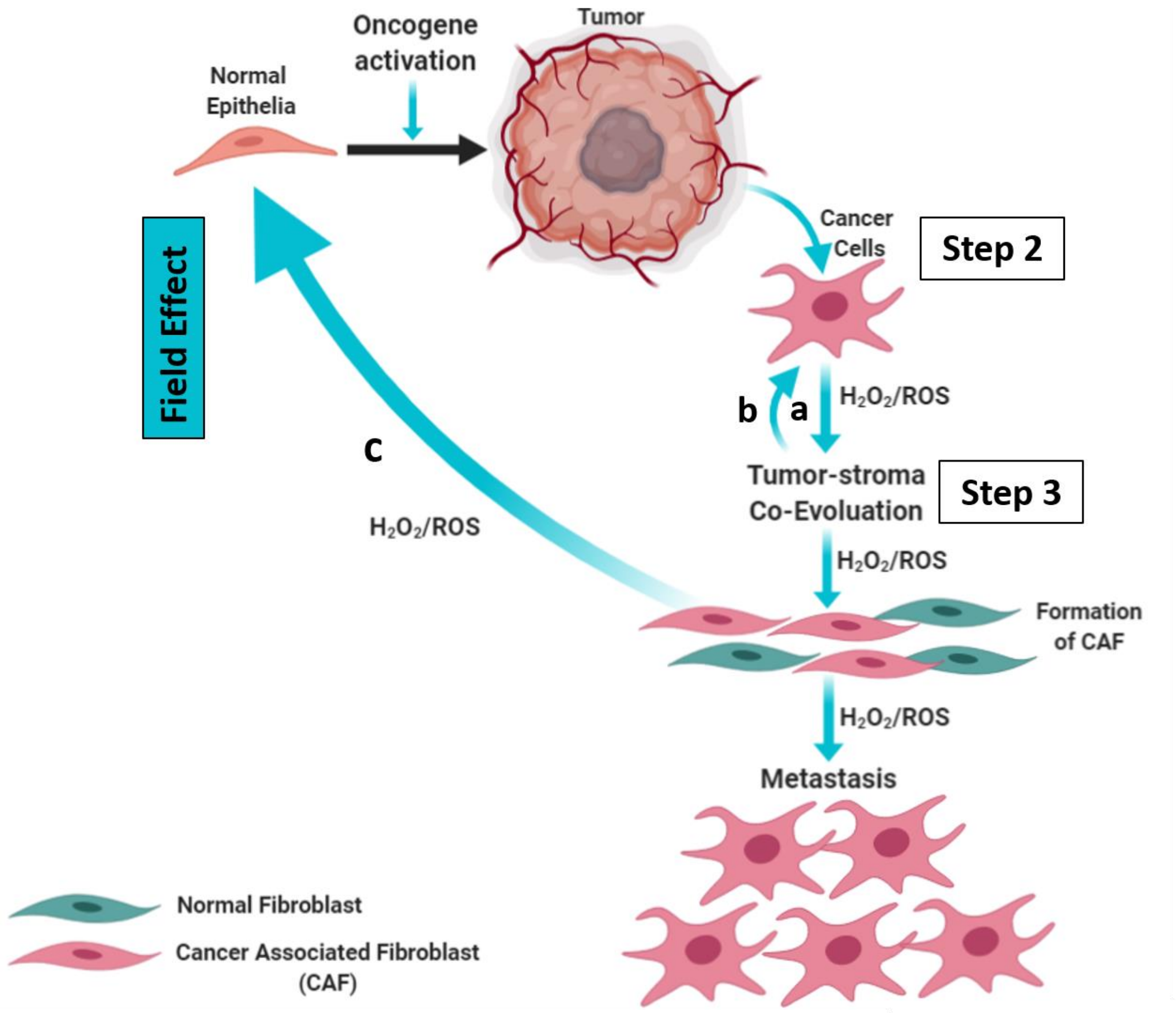

Figure sup19.Tumor Progression and Metastasis (steps 2 and 3). Once cancer cells are formed via oncogene activation in normal epithelial cells, then these cancer cells also begin to produce and secrete hydrogen peroxide (Step 2) to promote tumor-stroma co-evolution and metastasis (Step 3). In part a, cancer cells secrete hydrogen peroxide, which induces oxidative stress in neighboring stromal cells, such as fibroblasts. In part b, oxidative stress in fibroblasts leads to ROS production in the tumor stroma, which then further mutagenesis the cancer cells, allowing them to evolve to a more aggressive state, driving stromal lactate production and metastasis. Finally, in part c, hydrogen peroxide and ROS production could also mutagenize adjacent normal epithelial cells, further driving the formation of new cancer cells. This step, part c, may also account for the “field effect”, in which an entire area of tissue appears normal, but has been “cancerized” by hydrogen peroxide, oxidative stress, and DNA damage [9].

Reference

[1] J. Zhang *et al.*, “Nondestructive tissue analysis for ex vivo and in vivo cancer diagnosis using a handheld mass spectrometry system,” *Sci. Transl. Med.*, vol. 9, no. 406, p. eaan3968, 2017.

[2] J. Zhang *et al.*, “Direct molecular analysis of in vivo and freshly excised tissues in human surgeries with the MasSpec Pen technology,” *medRxiv*, 2020.

[3] M. Thill, “MarginProbe®: intraoperative margin assessment during breast conserving surgery by using radiofrequency spectroscopy,” *Expert Rev. Med. Devices*, vol. 10, no. 3, pp. 301–315, 2013.

[4] N. L. Martirosyan *et al.*, “Prospective evaluation of the utility of intraoperative confocal laser endomicroscopy in patients with brain neoplasms using fluorescein sodium: experience with 74 cases,” *Neurosurg. Focus*, vol. 40, no. 3, p. E11, 2016.

[5] J. D. Seidman, M. Ashton, and M. Lefkowitz, “Atypical apocrine adenosis of the breast: A clinicopathologic study of 37 patients with 8.7-year follow-up,” *Cancer Interdiscip. Int. J. Am. Cancer Soc.*, vol. 77, no. 12, pp. 2529–2537, 1996.

[6] T. M. A. Abdel-Fatah, D. G. Powe, Z. Hodi, A. H. S. Lee, J. S. Reis-Filho, and I. O. Ellis, “High frequency of coexistence of columnar cell lesions, lobular neoplasia, and low grade ductal carcinoma in situ with invasive tubular carcinoma and invasive lobular carcinoma,” *Am. J. Surg. Pathol.*, vol. 31, no. 3, pp. 417–426, 2007.

[7] D. J. Dabbs, G. Carter, M. Fudge, Y. Peng, P. Swalsky, and S. Finkelstein, “Molecular alterations in columnar cell lesions of the breast,” *Mod. Pathol.*, vol. 19, no. 3, pp. 344–349, 2006.

[8] S. Björner *et al.*, “Epithelial and stromal microRNA signatures of columnar cell hyperplasia linking Let-7c to precancerous and cancerous breast cancer cell proliferation,” *PLoS One*, vol. 9, no. 8, p. e105099, 2014.

[9] M. P. Lisanti *et al.*, “Hydrogen peroxide fuels aging, inflammation, cancer metabolism and metastasis: the seed and soil also needs" fertilizer",” *Cell cycle*, vol. 10, no. 15, pp. 2440–2449, 2011.

[10] F. Lozy and V. Karantza, “Autophagy and cancer cell metabolism,” in *Seminars in cell & developmental biology*, 2012, vol. 23, no. 4, pp. 395–401.
