# Supplementary material for: Human study on cancer diagnostic probe (CDP) for real‐time excising of breast positive cavity side margins based on tracing hypoxia glycolysis; checking diagnostic accuracy in non‐neoadjuvant cases
Source: Cancer Med. 2022 Feb 28;11(7):1630–45. doi: 10.1002/cam4.4503 (PMC8986141; doi:10.1002/cam4.4503)
Supplement: Supplementary file 1 — Supplementary Material [file CAM4-11-1630-s001.zip › cam44503-sup-0005-Supinfo.pdf]

Tehran University of Medical Sciences

Vice-Chancellor in Research Affairs- Tehran University of Medical Sciences

Research Ethics Certificate

Approval ID:	IR.TUMS.VCR.REC.1397.355	Approval Date:	2018-08-18
Evaluated by:	Vice-Chancellor in Research Affairs- Tehran University of Medical Sciences		
Status:	Approved		
Approval Statement:	The project was found to be in accordance to the ethical principles and the national norms and standards for conducting Medical Research in Iran.<br>Notice:<br>1. Although the proposal has been approved by the research ethics committee, meeting the professional and legal requirements is the sole responsibility of the PI and other project collaborators.<br>2. This certificate is reliant on the proposal/documents received by this committee on 2018-08-18. The committee must be notified by the PI as soon as the proposal/documents are modified.		
Proposal Title:	Clinical trial of fast (real-time) diagnostic probe about cancerous nature of suspicious regions in live tissue media during surgery surgery by electrochemical monitoring of Hypoxia glycolysis		
Principal Investigator:	Name: Mohammad Abdolahad<br>Email: m.abdolahad@ut.ac.ir		

Dr. Mohammad Ali Sahraian
Director of Institutional Research Ethics Committee
Vice-Chancellor in Research Affairs- Tehran University of Medical Sciences

Dr. Shokoufeh Nikfar
Secretary of Institutional Research Ethics Committee
Vice-Chancellor in Research Affairs- Tehran University of Medical Sciences
