# Supplementary material for: Human study on cancer diagnostic probe (CDP) for real‐time excising of breast positive cavity side margins based on tracing hypoxia glycolysis; checking diagnostic accuracy in non‐neoadjuvant cases
Source: Cancer Med. 2022 Feb 28;11(7):1630–45. doi: 10.1002/cam4.4503 (PMC8986141; doi:10.1002/cam4.4503)
Supplement: Supplementary file 1 — Supplementary Material [file CAM4-11-1630-s001.zip › cam44503-sup-0001-Supinfo.pdf]

Clinical Trial Protocol
Iranian Registry of Clinical Trials

20 Feb 2021

Real-time intra-operative in-vivo diagnosis of internal margins involved to breast pre-invasive/invasive cancer cells through pathological classification using a handheld electrochemical system

Protocol summary

Study aim

Checking the surgical margins in breast cancer patients is a critical step to be ensured from safe removal of high-risk suspicious cells with minimal dissection of normal tissues. Remained neoplastic lesions inside the body, induces unavoidable re-surgery and post-surgical therapies which cause many side effects. Here we introduce a real-time intra-operative breast margin checking tool with the capability of diagnosing the pathological state of the tissues. This probe (Cancer Diagnostic Probe CDP) comprises three integrated sensing needles and an electrical readout board. We carried out the diagnosis by entering the needles in the margin tissue followed by recording and compiling the electrochemical peak data in less than 40 seconds.

Design

A pragmatic, cancer patients community-based, single group, randomized trial

Settings and conduct

Frozen and permanent are being done as a routine procedure for every patient during surgery. If a region scored positive by CDP, its adjacent should also be checked. CDP dissected samples are compared to both permanent and frozen of reciprocal external margins of the patients. During surgery, the pathologist opinion about involved external margins in the frozen section is considered as the final diagnosis.

Participants/Inclusion and exclusion criteria

We select patients from all categories of breast cancer candidate for mastectomy or lumpectomy.

Intervention groups

During breast surgery after tumor dissection, CDP checks all the internal margins and the legions checked will be dissected either scored positive or negative by CDP and will be se for permanent pathology as CDP samples.

Main outcome variables

Intra-operative, non- invasive pathological classification of internal margins, in correlation with the metabolisms of breast cells, is the main achievement of CDP as a real-time diagnostic tool.

General information

Reason for update

Acronym

CDP, Cancer Diagnostic probe

IRCT registration information

IRCT registration number: **IRCT20190904044697N1**
Registration date: **2019-10-01, 1398/07/09**
Registration timing: **registered_while_recruiting**

Last update: **2019-10-01, 1398/07/09**
Update count: **0**

Registration date

2019-10-01, 1398/07/09

Registrant information

Name

Mohammad Abdolahad

Name of organization / entity

Country

Iran (Islamic Republic of)

Phone

+98 21 8802 8367

Email address

m.abdolahad@ut.ac.ir

Recruitment status

Recruitment complete

Funding source

Expected recruitment start date

2018-08-25, 1397/06/03

Expected recruitment end date

2019-12-21, 1398/09/30

Actual recruitment start date

empty

Actual recruitment end date
empty

Trial completion date
empty

Scientific title
Real-time intra-operative in-vivo diagnosis of internal margins involved to breast pre-invasive/invasive cancer cells through pathological classification using a handheld electrochemical system

Public title
Intra-operative real-time diagnostic probe for suspicious legions using electrochemical tracing of hypoxia glycolysis

Purpose
Diagnostic

Inclusion/Exclusion criteria

Inclusion criteria:
Patients with obvious breast tumor mass, previously diagnosed with solid mass (DCIS, ILC, IDC...). Neo-adjuvant cases with or without a wire guide.

Exclusion criteria:
No exclusion regarding patients recruitment

Age
No age limit

Gender
Both

Phase
N/A

Groups that have been masked
No information

Sample size
Target sample size: **200**
More than 1 sample in each individual
Number of samples in each individual: **6**
There are 6 distinguished margins in both tumor side (external margin) and body side (internal margin) included: superior, inferior, medial, lateral, superficial and deep. For each patient at least these margins should be tested.

Randomization (investigator's opinion)
Randomized

Randomization description
We should select almost 200 patients from all categories of breast cancer for in-vivo tests. Among all types of breast cancers, patients with IDC tumors are more than other types (such as phylloids and lobular carcinomas). Our surgical collaborators randomly select and introduce the patients for this trial. Each patient who accepts to take part in the investigation will sign ethical consent. The number of patients needed for this project was determined by the ethics committee. However, we took permission to investigate more patients to ensure the reliability and repeatability of our results.

Blinding (investigator's opinion)
Not blinded

Blinding description

Placebo
Not used

Assignment
Single

Other design features
The system lively determines the H2O2 released from cancer or atypical cells, through reverse Warburg effect and hypoxia assisted glycolysis pathways, in a quantitative electrochemical manner. We proposed a matched clinical diagnostic categorization between the pathological results of the tested tissues and response peaks of CDP based on ductal intraepithelial neoplasia (DIN) classification (with the latest reported modifications) based on our primary outcome results. Unique ability in the non-invasive and real-time diagnosis of internal margins with pathological values (from high-risk benign to pre-invasive and invasive cancer lesions) makes CDP a distinct intra-operative tool with simple and small handheld equipment to increase the prognostic factor of the cancer patients.

Secondary Ids

empty

Ethics committees

1

Ethics committee

Name of ethics committee
Ethics committee of Tehran University of Medical Science

Street address
No.23, 16 Azar Ave, Tehran Ave., Tehran, Iran Postal Code: 1417863181

City
Tehran

Province
Tehran

Postal code
1417863181

Approval date
2018-08-18, 1397/05/27

Ethics committee reference number
IR.TUMS.VCR.REC.1397.355

Health conditions studied

1

Description of health condition studied
Breast cancer surgery

ICD-10 code
C50

ICD-10 code description
Malignant neoplasm of breast, Breast cancer surgery, Hyperplasia

Primary outcomes

1

Description
Pathological classification of different breast lesions

correlated with current peaks of CDP. Results show meaningful consistency between DIN (Ductal Intraepithelial Neoplasia) based pathological diagnosis and CDP scoring.

Timepoint
Results will be compared with permanent pathology about 2 weeks after intervention

Method of measurement
Electrochemical Cyclic voltammetry

Secondary outcomes

empty

Intervention groups

1

Description
Intervention group: After tumor dissection, all the regions in body side margins named internal margins are tested by CDP. Depend on the size of the tumor and its proximity to one of the margins (not all the margins), some margins must undergo further analysis. The internal regions with more joint boundaries with the tumors would require further scans because of their larger formed internal margins. The head probe needles are single-used and the entered length of the needles into the breast margins are 4 millimeters. Scan region is 3*3*4 mm3 and if CDP score is positive, this area will be dissected for pathological examination.

Category
Diagnosis

Recruitment centers

1

Recruitment center
Name of recruitment center
Noor afshar hospital
Full name of responsible person
Dr Mohammad Abdolahad
Street address
Sadeghin Alley (17th West), Khodaverdi St, Pourebtehaj (Kashanak) St, Bahonar (Niavaran) Sq, Tehran, Iran
City
Tehran
Province
Tehran
Postal code
1978734763
Phone
+98 21 2282 4069
Fax
+98 21 2282 4060
Email
m.abdolahad@ut.ac.ir
Web page address
https://noor-afshar.ir

2

Recruitment center
Name of recruitment center
Imam Khomeini hospital
Full name of responsible person
Dr Mohammad Abdolahad
Street address
Imam Khomeini Hospital Complex, End of Keshavarz Blvd, Tehran, Iran
City
Tehran
Province
Tehran
Postal code
1419733141
Phone
+98 21 6119 0000
Email
m.abdolahad@ut.ac.ir
Web page address
http://ikhc.tums.ac.ir

3

Recruitment center
Name of recruitment center
Motamed cancer institute, Breast cancer research center
Full name of responsible person
Dr Mohammad Abdolahad
Street address
No. 45, Shahid Nazari Avenue, Aboureihan street, Enghelab square, Tehran, Iran
City
Tehran
Province
Tehran
Postal code
1315685981
Phone
+98 21 8887 6869
Email
Info@ibcrc.ir
Web page address
http://ibcrc.ir

4

Recruitment center
Name of recruitment center
Khatam Ol Anbia hospital
Full name of responsible person
Dr Mohammad Abdolahad
Street address
Vali Asr Ave, Rashid Yasemi Street, Tehran, Iran
City
Tehran
Province
Tehran
Postal code
1996835911
Phone

+98 21 8888 4040
Email
m.abdolahad@ut.ac.ir
Web page address
https://www.khatamhospital.org

5

Recruitment center
Name of recruitment center
Bozorgmehr Limited Surgery Center (Di clinic)
Full name of responsible person
Dr Mohammad Abdolahad
Street address
No. 51, Bozorgmehr St, Enghelab St, Tehran, Iran
City
Tehran
Province
Tehran
Postal code
1417935793
Phone
+98 21 6641 2697
Email
publicrel@ut.ac.ir
Web page address

Sponsors / Funding sources

1

Sponsor
Name of organization / entity
Iran Nano Fund
Full name of responsible person
Dr Mohammad Hosein Bahreini
Street address
No. 38, West Nastaran Ave (Arab), Khorramshahr St., North Sohrevardi St., Tehran
City
Tehran
Province
Tehran
Postal code
1533984611
Phone
+98 21 8876 9188
Email
info@nanofund.ir
Web page address
http://nanofund.ir
Grant name
Grant code / Reference number
Is the source of funding the same sponsor organization/entity?
Yes
Title of funding source
Iran Nano Fund
Proportion provided by this source
70
Public or private sector
Private
Domestic or foreign origin
Domestic
Category of foreign source of funding
empty
Country of origin
Type of organization providing the funding
Academic

Person responsible for general inquiries

Contact
Name of organization / entity
Tehran University of Medical Sciences
Full name of responsible person
Dr Mohammad Abdolahad
Position
Associate professor
Latest degree
Ph.D.
Other areas of specialty/work
Breast cancer surgery
Street address
Nano bio electronic lab, ground floor, school of electrical and computer engineering, university of Tehran faculty of engineering, North Kargar Ave.
City
Tehran
Province
Tehran
Postal code
1439957131
Phone
+98 21 8802 8367
Email
m.abdolahad@ut.ac.ir
Web page address
https://nbel.ut.ac.ir

Person responsible for scientific inquiries

Contact
Name of organization / entity
Tehran University of Medical Sciences
Full name of responsible person
Dr Mohammad Abdolahad
Position
Associate professor
Latest degree
Ph.D.
Other areas of specialty/work
Breast cancer surgery
Street address
Nano bio electronic lab, ground floor, school of electrical and computer engineering, university of Tehran faculty of engineering, North Kargar Ave
City
Tehran
Province
Tehran
Postal code
1439957131

Phone
+98 21 8802 8367
Email
m.abdolahad@ut.ac.ir
Web page address
http://nbel.ut.ac.ir

Person responsible for updating data

Contact
Name of organization / entity
Tehran University of Medical Sciences
Full name of responsible person
Dr Mohammad Abdolahad
Position
Associate professor
Latest degree
Ph.D.
Other areas of specialty/work
Breast cancer surgery
Street address
Nano bio electronic lab, ground floor, school of electrical and computer engineering, university of Tehran faculty of engineering, North Kargar Ave.
City
Tehran
Province
Tehran
Postal code
1439957131
Phone
+98 21 8802 8367
Email
m.abdolahad@ut.ac.ir
Web page address
http://nbel.ut.ac.ir

Sharing plan

Deidentified Individual Participant Data Set (IPD)
Yes - There is a plan to make this available
Study Protocol
Yes - There is a plan to make this available
Statistical Analysis Plan
Not applicable
Informed Consent Form
Yes - There is a plan to make this available
Clinical Study Report
Yes - There is a plan to make this available
Analytic Code
Not applicable
Data Dictionary
Not applicable
Title and more details about the data/document
60% of the information about the main outcome, can be shared.
When the data will become available and for how long
Starting 6 months after publication.
To whom data/document is available
The data will be available for medical staff and academic institutions.
Under which criteria data/document could be used
Non-identifiable personal data will not be usable for the applicant and will only inform the patient of a positive clinical trial that the device is functioning properly.
From where data/document is obtainable
Applicants can request data access via email. The data will be available to the applicant in a categorized manner with pathology reports.
What processes are involved for a request to access data/document
We will send the data to the applicant in 2 weeks.
Comments
